# Sema3d Restrained Hepatocellular Carcinoma Progression Through Inactivating Pi3k/Akt Signaling *via* Interaction With FLNA

**DOI:** 10.3389/fonc.2022.913498

**Published:** 2022-07-25

**Authors:** Yiming Li, Cong Xu, Bo Sun, Fangjing Zhong, Momo Cao, Lianyue Yang

**Affiliations:** Liver Cancer Laboratory, Department of Surgery, Xiangya Hospital, Central South University, Changsha, China

**Keywords:** Sema3d, semaphorin 3d, hepatocellular carcinoma, prognosis, cancer progression, Pi3k/Akt signaling, EMT

## Abstract

Hepatocellular carcinoma (HCC) is one of the most lethal malignant tumors worldwide due to the high incidence rate of metastasis and recurrence. Semaphorin 3d (Sema3d) has been shown to play a critical role in vascular development during early embryogenesis and several forms of cancer progression *via* regulating cell migration. However, the function of Sema3d in hepatocellular carcinoma (HCC) remains elusive. This study aimed to explore the function and mechanisms of Sema3d in HCC. In our study, Sema3d expression was significantly downregulated in HCC tissues and cell lines. Downregulated Sema3d was closely correlated with aggressive clinicopathological features and poor clinical outcomes in HCC patients. Moreover, overexpression of Sema3d in HCCLM3 cells was significantly inhibited and knockdown of Sema3d in PLC/PRF/5 cells promoted proliferation, migration, invasion, and epithelial–mesenchymal transition (EMT) of HCC cells *in vitro* and tumor growth, EMT, and metastasis *in vivo*. Furthermore, the RNA sequencing and gene set enrichment analysis (GSEA) indicated that these phenotypic and functional changes in Sema3d-interfered HCC cells were mediated by the Pi3k/Akt signaling pathway, and co-IP–combined mass spectrometry indicated Sema3d might interact with FLNA. Finally, we proved that Sema3d exerted its tumor-restraining effect by interacting with FLNA to inactivate the Pi3k/Akt signaling pathway and remodel the cytoskeleton. Our data showed that Sema3d restrained hepatocellular carcinoma proliferation, invasion, and metastasis through inactivating Pi3k/Akt *via* interaction with FLNA, which may serve as a novel prognostic predictor and a potential therapeutic target for HCC patients.

## Introduction

In 2018, liver cancer was predicted to cause 84 million new cases and 78 million deaths worldwide, ranking it sixth most commonly diagnosed and fourth most lethal cancer, with hepatocellular carcinoma (HCC) accounting for 75%–85% of all cases (1). The mainly curative methods for early-stage tumors are liver resection, liver transplantation, or local ablation, but these methods remain unsatisfactory for most HCC patients due to the relatively high incidence rate of metastasis and recurrence after treatment (2). The 5-year survival rate in HCC patients beyond the Milan criteria after surgical resection is only 30% to 50% (3, 4), and recurrence of HCC in the liver reaches 70% 5 years after resection (5). There are major unmet needs in HCC management through discovering new functional biomarkers, exploring mechanisms, and interfering with signaling cascades (6). As yet, many factors have been identified to play an important role in HCC progression, including aberrantly expressed miRNAs, LncRNAs, and proteins that have demonstrated potential value as HCC prognostic or diagnostic markers (7–10).

Class 3 semaphorins (Sema3 proteins) are a family of secreted and transmembrane signaling molecules (11) that have emerged as cancer biomarkers and critical regulators of growth, metastasis, and drug resistance (12–14). Semaphorin 3d (Sema3d) encodes a related member of this class of proteins and was originally found to be an axon guidance protein (15), and in succession, has also been shown to be particularly important in numerous and diverse biological processes, such as cardiovascular development, immune cell regulation, and tumor progression (16–18). For instance, Sema3d functions as a repellent guidance molecule for endothelial cells (11) and negatively regulates parathyroid cell growth *via* inhibited EGFR signaling (19). However, the knowledge about Sema3d in HCC was rarely discovered. Sema3d’s involvement in cell proliferation and migration gives rise to a hypothesis that Sema3d might play an important role in HCC progression.

In this study, the function of Sema3d was explored in HCC and found that downregulated Sema3d is closely correlated with poor prognosis of HCC patients. We also confirmed that downregulated Sema3d promotes HCC progression. Mechanism studies showed that Sema3d could inactivate the Pi3k/Akt signaling pathway and remodel the cytoskeleton *via* interaction with FLNA in HCC. Thus, Sema3d might serve as a potential prognostic biomarker and therapeutic target for HCC.

## Materials and Methods

### HCC Samples and Patients

The present study was approved by the Ethics Committee of Xiangya Hospital, Central South University. All patients and their families provided written informed consent and agreed to the use of their tissue samples in the study following the Declaration of Helsinki. A total of 180 HCC specimens in the training cohort collected from January 2013 to December 2015 were randomly selected from the patients who received liver resection at the Department of Surgery, Xiangya Hospital of Central South University. Another 120 HCC specimens in the validation cohort collected from January 2012 to December 2015 were randomly selected from the Department of Abdominal Surgical Oncology, Hunan Cancer Hospital. The patients’ demographics and clinicopathological variables of the two cohorts are described in the **Supplementary Tables**. Furthermore, 50 matched fresh HCC tissues and adjacent nontumoral liver tissues (ANLTs) were collected from Xiangya Hospital from June to September 2020. The diagnosis of HCC in all patients was confirmed by two independent histopathologists.

### Cell Lines and Cell Culture

MHCC97-L, MHCC97-H, and HCCLM3 were kindly provided by the Liver Cancer Institute of Fudan University, Shanghai, China. The PLC/PRF/5, Hep3B, and HepG2 cells were purchased from the American Type Culture Collection (ATCC, Manassas, VA, USA). Primary human hepatocytes (PHHs), SMMC7721, and Huh7 cells were purchased from the Cell Bank of the Typical Culture Preservation Committee of the Chinese Academy of Science, Shanghai, China. Cells were maintained in Dulbecco’s modified Eagle’s medium (Biological Industries, Kibbutz Beit HaEmek, Israel) supplemented with 10% fetal bovine serum, 100 μg/ml of streptomycin, and 100 U/ml of penicillin (Hyclone, Logan, UT, USA) at 37°C in a humidified atmosphere with 5% CO_2_.

### Protein Extraction and Western Blot

Tissues or cells were lysed with RIPA buffer (Pierce, Rockford, IL, USA) supplemented with 1% protease inhibitors. The lysates were centrifuged and then supernatants were collected. Nuclear and cytosol extractions were conducted using a Nuclear/Cytosol Fractionation Kit (K26625, BioVision, CA, USA) according to the protocols provided by the manufacturer. Protein concentration was measured using a BCA protein assay (Thermo Scientific, Rockford, IL, USA). Protein lysates, suspended in loading buffer, and an equal amount of 20 μg protein were separated on 10% or 8% SDS-polyacrylamide gels (depending on the molecular weight of detected protein) and transferred onto PVDF membranes (Millipore, Belford, MA, USA). These membranes were then blocked with 5% skim milk at room temperature for 1 h and incubated with primary antibodies at 4°C overnight. After being washed 3 times in PBST, they were incubated with suitable HRP-conjugated secondary antibody at room temperature for 30 min and washed 3 × 10 min, then detected using an enhanced chemiluminescence (ECL) kit (Thermo Scientific, MA, USA). Primary antibodies for Sema3d, FLNA, p-FLNA (S2125), C-FLNA (FLNA C-terminal), and p-AKT were obtained from Abcam (Cambridge, MA, USA), while those for E-cadherin, vimentin, Snail, AKT, Pi3k, and p-Pi3k were purchased from Cell Signaling Technology (MA, USA). The loading control protein, Lamin B1, β-actin, and GAPDH antibody were purchased from Affinity (Beijing, China). Details of the reagents are shown in **Supplementary Materials and Methods**.

### Immunohistochemical Analysis and Scoring

Immunohistochemical staining on formalin-fixed, paraffin-embedded tissue sections 5 μm in thickness was performed using the polymer HRP detection system (Zhongshan Goldenbridge Biotechnology, Beijing, China). Immunohistochemical experiments were conducted as previously described (10, 20). The sections were deparaffinized in xylene, rehydrated in a series of graded ethanols (100%, 95%, 75%, and 50% in sequence), and then antigen repaired in boiled sodium citrate solution. The endogenous peroxidase activity of hydrogen was blocked by peroxide for 30 min and antigen blocked by goat serum for 30 min at room temperature. The corresponding primary antibody was incubated at 4°C overnight. After being washed, tissue sections were incubated with HRP anti-rabbit IgG at room temperature for 30 min, followed by being incubated with DAB solution at the same time and then counterstained with hematoxylin. The immunohistochemical (IHC) score of target proteins was independently evaluated by two investigators according to the proportion and intensity of positive cells within five randomly selected fields per slide (magnification, ×400). The intensity was assessed by four grades: 0 for none; 1 for weak; 2 for moderate; and 3 for strong. The percentage of positive cells was divided into five degrees: 0, no positive tumor cells; 1 for ≤5%; 2 for 6%–25%; 3 for 26%–75%; and 4 for ≥76%. The immunoreactive score was calculated by multiplying the staining extent score by the intensity score. As previously reported, high expression was defined as a staining index score of >4, while low expression was defined as a staining index score of ≤4 (21, 22).

### 5-Ethynyl-2′-Deoxyuridine Proliferation Assay

The 5-ethynyl-2′-deoxyuridine (EdU) proliferation kit was purchased from RiboBio (Guangzhou, China), and the assays were carried out according to the protocol. The HCC cells were seeded in 96-well plates at a density of 5,000 cells/well. After adherence 24 h later, the medium was replaced by the ready-to-use EdU staining mixture medium that was made as per the manufacturer’s protocol, and then incubated for about 60 min. The cells were then washed with 0.5% Triton X-100 for 10 min × 3 times before being fixed with 4% formaldehyde for 15 min. Finally, cells were exposed to the Apollo reaction cocktail for 30 min and visualized under an inverted fluorescence microscope DMI4000-B (Leica, Wetzlar, Germany).

### Wound-Healing and Transwell Invasion Assays

For the wound-healing assay, 5 × 10^5^ cells were seeded into 6-well plates and grown to confluence. Mitomycin C (10 μg/ml) was used to suppress cell proliferation before scratching. Wounds were created by scraping the confluent cell monolayers with a 10-μl pipette tip. After being extensively rinsed to remove cellular debris, the cells were cultured in a serum-free medium. The wound closure rate was monitored every 12 h, and images were taken using an inverted microscope TE-2000S (Nikon, Tokyo, Japan). A Transwell invasion assay was performed in a 24-well Transwell plate with 8-μm polyethylene terephthalate membrane filters (Corning Costar Corp, Corning, NY, USA); 1 × 10^5^ cells in 200 μl of serum-free medium were added to the upper chambers, which contained Matrigel-coated membranes (BD Biosciences). Each lower chamber was filled with a 500-μl medium with 10% FBS. After 18 or 24 h of incubation, cells that invaded the bottom chamber were fixed with 4% paraformaldehyde and stained with 0.1% crystal violet. Invasive cells were counted in five randomly chosen fields (magnification, ×200) per well.

### HCC Mouse Models

Animal xenograft assays were conducted with 6-week-old male BALB/c nude mice (six mice per group); 5 × 10^6^ indicated cells were subcutaneously injected into the right dorsal flank region of nude mice. Tumor sizes were measured at the indicated time points and calculated with the following formula: Tumor volume = *L* × *W* ^2^ × 0.5 (*L*, length; *W*, width) (9). After 4 weeks, the mice were sacrificed, and the tumors were harvested to weigh and undergo further experiments. The subcutaneous tumor was then cut into pieces of the same size as 1 mm^3^ and implanted into the left liver of each group to mimic the primary HCC (6 in each group). Orthotopic tumor implantation was performed as described previously (8). The growth of liver orthotopic tumors was surveyed weekly by the *in vivo* lumina imaging system (IVIS, Caliper Life Sciences, Hopkinton, MA, USA). After 6 weeks, the mice were sacrificed, and the livers and lungs were harvested. The tumor size was measured by the vernier caliper as previously described (9), imaged, and processed for histopathological examination. All animal experiments were conducted at the Animal Institute of CSU according to the protocols approved by the Medical Experimental Animal Care Commission of CSU. As the proposal of the Animal Institute of CSU, the euthanasia of all the experimental mice was used the pentobarbital sodium, 150mg/Kg, and intraperitoneal injection.

### Statistical Analysis

Statistical analysis was performed using the SPSS 24.0 software (SPSS Inc., Chicago, IL, USA). The experimental data were presented as the mean ± SD and analyzed using Student’s *t*-test. The Chi-squared test was applied to examine the association between Sema3d expression and clinicopathological parameters. Survival curves for patients were calculated using the Kaplan-Meier method and analyzed using the log-rank test. Prognostic factors were examined by univariate and multivariate analyses using the Cox proportional hazards model. Spearman’s rank analysis was performed to determine the correlation between different protein levels. One-sample *t*-test or Wilcoxon signed-rank test was performed to measure the membrane and cytolytic protein expression level. All differences were deemed statistically significant at *p* < 0.05.

Further details of *Materials and Methods*. are described in the **Supplementary Materials and Methods**.

## Results

### Sema3d Is Downregulated in HCC Cell Lines and Tissues

**Figure 1:**
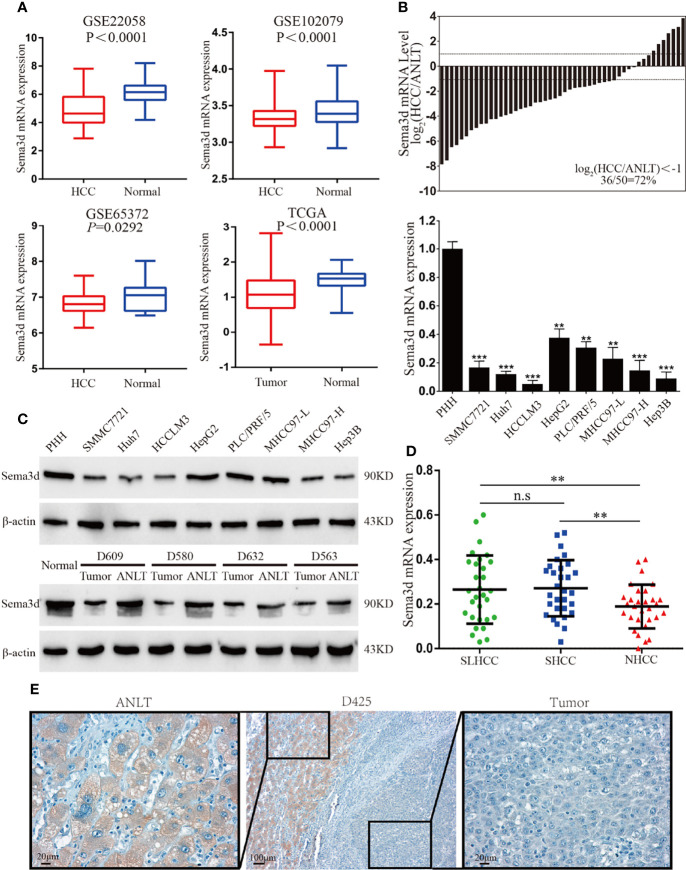
Sema3d is downregulated in HCC cell lines and tissues. **(A)** Sema3d expression in Gene Expression Omnibus (GEO) datasets (GSE22058, GSE102079, GSE56372) and TCGA database. **(B)** Sema3d mRNA expression level was detected by qRT-PCR in 50 HCC and paired ANLT samples (upper panel), 8 HCC cell lines, and primary human hepatocytes (PHHs) (lower panel). **(C)** Sema3d protein expression was examined by Western blot in PHHs and HCC cell lines, representative normal liver tissue, paired HCC tissues, and ANLTs. **(D)** Sema3d mRNA expression in HCC tissues of 3 clinical subtypes (SLHCC, SHCC, and NHCC) was detected by qRT-PCR. **(E)** Representative IHC images of Sema3d expression in HCC tissues and corresponding ANLTs. ^**^ *p* < 0.01; ^***^ *p* < 0.001. n.s, no significance.

Firstly, we compared the Sema3d mRNA expression levels of HCC and normal tissues from the Gene Expression Omnibus datasets (**Figure 1A**, GSE22058, GSE102079, GSE56372) and TCGA database, revealing that Sema3d is downregulated in HCC. We then examined the expression of Sema3d mRNA in 50 pairs of frozen HCC tissues and the corresponding adjusted nontumor liver tissues (ANLTs), and Sema3d mRNA was expressed lower in 72% of HCC tissues than in the corresponding ANLTs. We also detected the mRNA expression levels of Sema3d mRNA in 8 HCC cell lines and 1 normal hepatocyte, the PHHs (**Figure 1B**). The protein expression level of Sema3d in tumor tissues and cell lines was detected by Western blot (**Figure 1C**), consistent with the GEO and TCGA datasets. The Sema3d expression level in HCC tissues and cells was significantly lower than ANLTs and normal hepatocytes. Previously, we defined a specific subtype of HCC as solitary large hepatocellular carcinoma (SLHCC) and divided HCC into three different clinical subtypes: SLHCC (single tumor, tumor diameter >5 cm), nodular HCC (NHCC, node number >1), and small HCC (SHCC, tumor diameter ≤5 cm). We demonstrated that SLHCC possessed unique clinicopathological and molecular pathological characteristics and exhibited a similar long-term overall and disease-free survival with SHCC but much better than NHCC (4, 10, 23, 24). We detected mRNA of Sema3d in the 3 clinical subtypes of HCC (**Figure 1D**), and the SLHCC and SHCC possessed low metastatic potentials and expressed relatively higher Sema3d than the NHCC with high metastatic potentials. The expression level of Sema3d was also analyzed by IHC and expressed lower in HCC than the relative ANLT (**Figure 1E**). These data reveal that Sema3d is downregulated in HCC cell lines and tissues, indicating that the role of downregulated Sema3d might be crucial in HCC progression.

### Downregulated Sema3d Is Associated With Poor Prognosis

**Figure 2:**
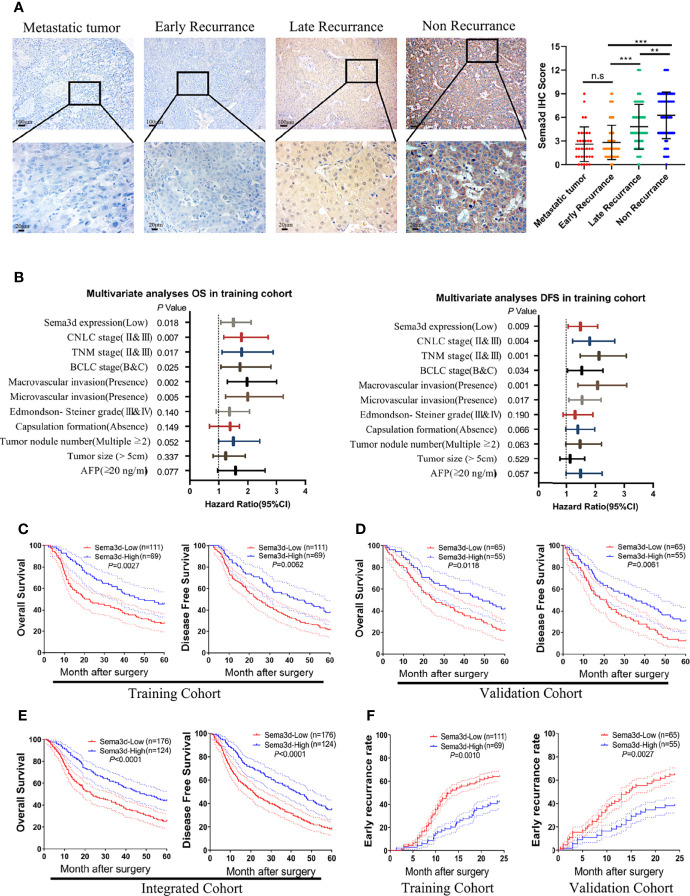
Downregulated Sema3d is associated with HCC’s poor prognosis. **(A)** Representative IHC staining for Sema3d in metastatic tumor (*n* = 34), early recurrence of HCC ( < 2 years, *n* = 142), late recurrence of HCC ( > 2 years, *n* = 66), and nonrecurrence of HCC (5 years, *n* = 92). IHC staining score of Sema3d in the integrated cohorts (composed of training and validation cohorts). *p*-values were calculated by the Mann–Whitney *U* test. ^**^ *p* < 0.01; ^***^ *p* < 0.001. n.s, no significance. **(B)** Multivariate analysis revealed that low Sema3d expression was an independent risk factor for overall survival (OS) and disease-free survival (DFS) in the training cohort. **(C)** Survival analysis for the training cohort reveals the Sema3d-low group had worse OS and DFS. **(D)** Survival analysis for the validation cohort verified that the Sema3d-low group had worse OS and DFS. **(E)** Survival analysis for integrated cohort (composed of training and validation cohorts) verified that the Sema3d-low group had worse OS and DFS. **(F)** Kaplan–Meier curves for the cumulative early recurrence ( < 2 years) rate of HCC patients based on Sema3d expression in training and validation cohorts.

To evaluate the correlations among Sema3d and clinicopathological variables, we analyzed Sema3d expression level by IHC and scored the expression intensity and percentage of positive cells in the training and validation cohorts. We found that Sema3d is low expressed in metastatic tumor and early recurrence (<2 years) of HCC tissues but relatively high expressed in late recurrence (>2 years) and nonrecurrence (in 60 months after surgery) of HCC tissues (**Figure 2A**). We then analyzed the association of Sema3d IHC scores with clinicopathological features and survival of HCC patients using the training cohort (*n* = 180) and validation cohort (*n* = 120). In the training and validation cohorts, a low expression level of Sema3d (IHC score ≤4) was closely correlated with tumor size, tumor nodular number, micro- and macrovascular invasion, advanced tumor node metastasis stage (TNM), Barcelona Clinic Liver Cancer (BCLC) stage, and China Clinic Liver Cancer (CNLC) stage (25) (all *p* < 0.05, **Supplementary Table S1**). Furthermore, uni- and multivariate analyses in the training cohort revealed that low Sema3d expression was an independent risk factor for both OS and DFS of HCC patients after liver resection (**Figure 2B**; **Supplementary Table S2**). Correspondingly, these results were further verified in the validation cohort (**Supplementary Table S3**). Notably, survival analysis for the training cohort reveals that HCC patients in the low Sema3d expression group had worse OS (1-, 3-, and 5-year OS: 88.41%, 59.06%, and 45.73% vs. 64.63%, 40.50%, and 27.66%) and DFS rates (1-, 3-, and 5-year DFS: 86.96%, 56.87%, and 37.68% vs. 63.06%, 36.94%, and 21.62%) than the patients in the high expression group (**Figure 2C**). The survival analysis for the validation cohort and the integrated cohort which concluded the two cohorts both confirmed the worse OS and DFS in low Sema3d group HCC patients (**Figures 2D, E**). Kaplan–Meier curves of the OS, DFS, and progression-free survival (PFS) in TCGA also indicated that the low Sema3d expression group had shorter OS, DFS, and PFS (**Supplementary Figure S1**). More than that, Kaplan–Meier curves for the cumulative early recurrence (<2 years) rate of HCC patients in the training and validation cohorts showed a higher early recurrence rate in the low Sema3d group (**Figure 2F**). These data fully confirmed that downregulated Sema3d was closely correlated with poor survival and could be used as a novel independent prognosis biomarker for HCC patients after hepatic resection.

### Sema3d Restrains HCC Migration, Invasion, Proliferation, and Metastasis *In Vitro* and *In Vivo*

**Figure 3:**
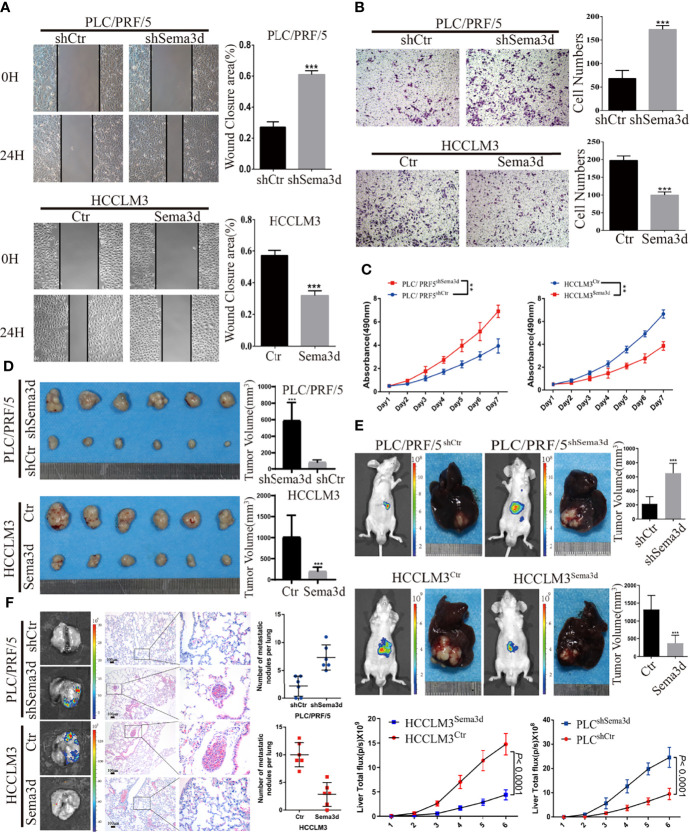
Sema3d restrains HCC migration, invasion, proliferation, and metastasis *in vitro* and *in vivo*. **(A)** Wound-healing assay detected the migratory PLC/PRF/5^shSema3d^ and HCCLM3^Sema3d^, as well as the corresponding control cells (*n* = 6 for each group). **(B)** A Transwell invasion assay was performed to detect the invasive capacities of HCC cells (*n* = 6 for each group). **(C)** MTT assay detected the proliferation capacities of Sema3d-interfered HCC cells (*n* = 6 for each group). **(D)** In subcutaneous tumors derived from PLC/PRF/5^shSema3d^ and HCCLM3^Sema3d^ cells and their control cells, tumor volumes were calculated with the formula (length × width^2^)/2. **(E)** At week 6, representative bioluminescent images monitored by IVIS and orthotopic xenograft tumors were shown, as well as the volume of orthotopic tumors and the growth curves constructed by the quantified bioluminescent signal (*n* = 6 for each group). **(F)** Representative bioluminescent images and HE staining images of lung tissue and a number of pulmonary metastasis nodules in different groups were shown (upper panel). ^**^ *p* < 0.01; ^***^ *p* < 0.001.

To understand the function of Sema3d in HCC cells, we stably overexpressed Sema3d in low-expressed HCCLM3 cells and knocked it down in high-expressed PLC/PRF/5 cells by lentivirus transfection. The expression of Sema3d in these resultant cells (HCCLM3^Ctr^, HCCLM3^Sema3d^ and PLC/PRF/5^shCtr^, PLC/PRF/5^ShSema3d^) was verified by qRT-PCR and Western blot (**Supplementary Figure S2**), and the most effective sequence was selected. The wound-healing and Transwell invasion assays were used to investigate migration and invasion capacity. The results showed that HCCLM3^Sema3d^ cells had a lower wound closure rate and less invasion than HCCLM3^Ctr^ cells, and PLC/PRF/5^ShSema3d^ had also markedly acquired migratory and invasive capacity than PLC/PRF/5^shCtr^ cells (**Figures 3A, B**). The MTT assay and EdU assay indicated that Sema3d inhibited proliferation of HCCLM3^Sema3d^ and PLC/PRF/5^shCtr^ cells (**Figure 3C**; **Supplementary Figure S3**). To verify the above findings *in vivo*, we established subcutaneous xenograft tumor and orthotopic xenograft tumor models, as previously described (26). After 4 weeks, HCCLM3^Ctr^ and PLC/PRF/5^ShSema3d^ cell-derived tumors at the SC implantation sites were larger and grew more rapidly than HCCLM3^Sema3d^ and PLC/PRF/5^shCtr^ cell-derived tumors (**Figure 3D**). Consistently, liver orthotopic xenograft tumor and IVIS imaging showed that tumors in HCCLM3^Sema3d^ had smaller volume and weaker fluorescence signals than those in the HCCLM3^Ctr^ group, and tumors from the PLC/PRF/5^shSema3d^ group exhibited larger volume and stronger fluorescence signals than those from the PLC/PRF/5^shCtr^ group (**Figure 3E**). The growth curves of orthotopic tumors were constructed by the quantified bioluminescent signal and revealed a higher growth rate in the PLC/PRF/5^shSema3d^ and HCCLM3^Ctr^ cell-derived tumors than in the PLC/PRF/5^shCtr^ and HCCLM3^Sema3d^. In the H&E staining of liver orthotopic xenograft tumors, we also noticed that the PLC/PRF/5^shSema3d^ and HCCLM3^Ctr^ cell-derived tumors exhibit the character of invasive extension with irregular borders and multinodularity, and the PLC/PRF/5^shCtr^ and HCCLM3^Sema3d^ cell-derived tumors demonstrated expansive growth with well-defined borders (**Supplementary Figure S4**). Moreover, *ex vivo* bioluminescent imaging and H&E staining of the lung both showed the incidence of lung metastasis was decreased in the HCCLM3^Sema3d^ group than in the HCCLM3^Ctr^ group but increased in the PLC/PRF/5^shSema3d^ group than the PLC/PRF/5^shCtr^ group (**Figure 3F**).

### Sema3d Restrains HCC Progression *via* Inactivating Pi3k/Akt Signaling

**Figure 4:**
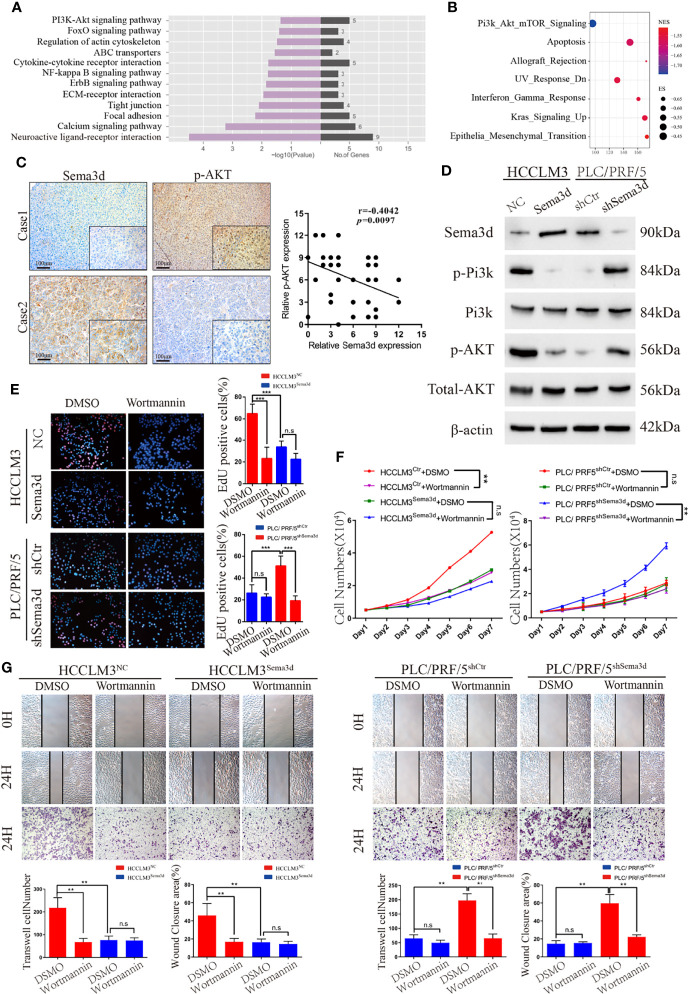
Sema3d restrains HCC progression *via* inactivating Pi3k/Akt signaling. **(A)** RNA-seq and KEGG analysis of HCCLM3^Sema3d^ cells compared with HCCLM3^NC^ cells revealed the potential signaling pathway regulated by Sema3d in HCC cells. **(B)** GSEA was performed between the Sema3d high- and low-expression patients in TCGA dataset. **(C)** Representative IHC images of Sema3d and p-AKT and their expression correlations were analyzed by Spearman’s rank correlation tests in 40 HCC tissue samples. **(D)** Expression of Pi3k and Akt and their phosphorylation level in Sema3d-interfered HCC cells were detected by Western blot. **(E)** EdU staining assay detected the proliferation for Sema3d-interfered HCC cells with/without Wortmannin treatment. **(F)** MTT assay detected the proliferation of ema3d-interfered HCC cells, and their control cells with/without Wortmannin treatment. **(G)** Wound healing and Transwell invasion assays detected the migration and invasion of Sema3d-interfered HCC cells with/without Wortmannin treatment. ^**^ *p* < 0.01; ^***^ *p* < 0.001. n.s, no significance.

We found that Sema3d restrained HCC growth and metastasis *in vitro* and *in vivo.* To elucidate the mechanism and screen the potential signaling manipulated by Sema3d, RNA-seq was utilized to compare the expression profiles between HCCLM3^Ctr^ and HCCLM3^Sema3d^ cells and demonstrated that 75 mRNAs upregulated and 282 mRNAs downregulated at least 2-fold change in HCCLM3^Sema3d^ cells (**Supplementary Figure S5**, the data uploaded at the GEO database: GSE200430). Subsequently, the KEGG analysis showed that several signaling was downregulated in HCCLM3^Sema3d^ cells (**Figure 4A**), and we also performed GSEA analysis in the Cancer Genome Atlas (TCGA) Liver Hepatocellular Carcinoma (LIHC) data (**Figure 4B**; **Supplementary Tables S5, S6**). Intriguingly, both of these two analyses point out that Pi3k/Akt signaling is significantly downregulated in the Sema3d high group, which indicates that Sema3d might affect the progression of HCC through Pi3k/Akt signaling (**Supplementary Figures S6, S7**).

It is also worth pointing out that Pi3k/Akt signaling is one of the most critical signals that control several kinds of cellular processes, including growth, metastasis, and is hyperactivated in approximately half of all HCC tumors (27, 28). We then detected Sema3d and phospho-Akt in 40 HCC tissues randomly selected in the training cohort by IHC (**Figure 4C**), and Spearman’s rank correlation test indicated the significant and negative correlation of phospho-Akt and Sema3d expression. We also detected Pi3k and Akt protein expression and their phosphorylation level in HCCLM3^Ctr^, HCCLM3^Sema3d^ and PLC/PRF/5^shCtr^, PLC/PRF5^shSema3d^ cells (**Figure 4D**). When we knockdown Sema3d in PLC/PRF5, the phosphorylation level of Pi3k and Akt increased but the total expression level was not affected, whereas ectopic expression of Sema3d in HCCLM3 decreased the phosphorylation level of Pi3k and Akt.

Furthermore, to confirm Pi3k/Akt signaling inactivated by Sema3d in HCC, the Sema3d-interfered HCC cells were treated with or without Wortmannin for 48 h, which was verified as a functional inhibitor of Pi3k/Akt signaling (29, 30). The MTT assay and EdU staining assay were performed to confirm that Wortmannin treatment obviously decreased the proliferation of Sema3d low-expressed PLC/PRF5^shSema3d^ and HCCLM3^Ctr^ cells, without a significant effect on the Sema3d high-expressed PLC/PRF/5^shCtr^ and HCCLM3^Sema3d^ cells (**Figures 4E, F**). The migration and invasion capacity were then detected by wound-healing and Transwell invasion assays (**Figure 4G**). Similarly, Wortmannin inhibited the migration and invasion of PLC/PRF5^shSema3d^ and HCCLM3^Ctr^ cells, with no obvious effect on Sema3d high-expressed cells. The above results indicated that Sema3d inhibited the progression of HCC cells *via* inactivating Pi3k/Akt signaling.

### Sema3d Directly Interacts With FLNA and Affects Cytoskeleton Remodeling

**Figure 5:**
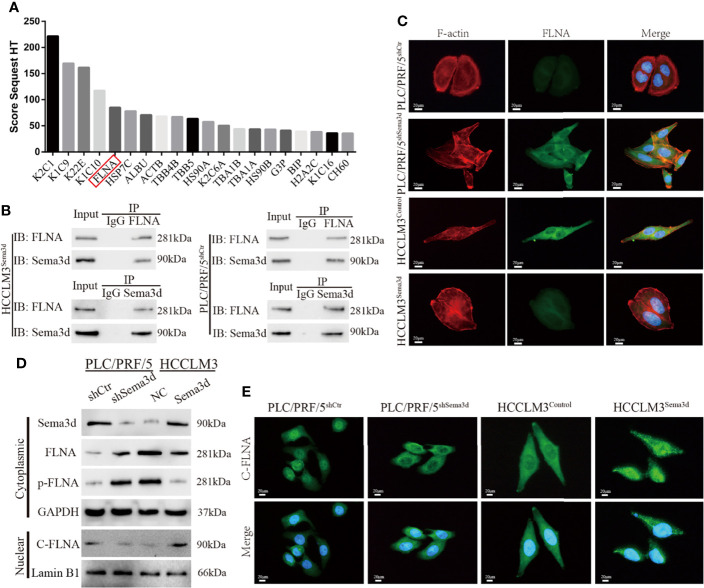
Sema3d directly interacts with FLNA and affects cytoskeleton remodeling. **(A)** Coimmunoprecipitation (co-IP) was performed to extract Sema3d direct combined proteins from HCCLM3^Sema3d^ cells, which were then identified using LC-MS/MS. The top 20 combined proteins sorted by Score Sequest are shown in the diagram. **(B)** co-IP was performed to determine that Sema3d and FLNA could directly interact with each other. **(C)** Double immunofluorescence staining showed that Sema3d affected the cellular expression of FLNA (green) and induced cytoskeleton remodeling (F-actin, red). **(D)** Protein expression levels were analyzed by Western blot and showed that Sema3d regulated the phosphorylation level of FLNA and the amount of nuclear C-FLNA. **(E)** Immunofluorescence staining revealed that Sema3d promotes C-FLNA translocated from the cytoplasm to the nucleus.

To address how Sema3d inactivates Pi3k/Akt signaling in Sema3d high-expressed PLC/PRF/5^shCtr^ and HCCLM3^Sema3d^ cells, we used coimmunoprecipitation with an anti-Sema3d antibody to coimmunoprecipitate endogenous Sema3d protein. The protein after coimmunoprecipitation was subjected to liquid chromatography-tandem mass spectrometry (LC-MS/MS) for protein identification. Finally, we have identified proteins ranked by score sequest, which indicates the potential capacity to combine with Sema3d protein (**Figure 5A**; **Supplementary Table S7**). In the potential interactors, FLNA has attracted our attention, which is a classical actin-crosslinking protein that stabilizes delicate three-dimensional actin networks during cell movements and has been identified as a regulator of Pi3k/Akt signaling in prostate tumors (31). Subsequently, co-IP results revealed that Sema3d could be directly combined with FLNA in HCC cells (**Figure 5B**).

In consideration of the intimate correlation of FLNA and cytoskeleton, we performed double immunofluorescence staining of Alexa Fluor phalloidin for F-actin and anti-FLNA in Sema3d interfered HCC cells. It is noteworthy that when we knockdown Sema3d in PLC/PRF5, the PLC/PRF5^shSema3d^ cells exhibited more F-actin fibers and changed from round-like epithelial morphology to spindle-like mesenchymal appearance, and the expression of FLNA also increased and colocalized with F-actin (**Figure 5C**). In contrast, overexpression of Sema3d induced the opposite result. The alteration of FLNA and F-actin indicates that Sema3d remodels the actin cytoskeleton through FLNA, and also reminded us that Sema3d might regulate EMT of HCC cells.

Previous studies have proven that the phosphorylation of FLNA could prevent the cleavage from a 280-kDa intact protein to a 90-kDa fragment and nucleus translocation (31, 32). To investigate whether Sema3d regulated FLNA in HCC cells, we detected full-length FLNA (280 kDa), phosphorylation of FLNA at Ser 2152, and nuclear 90 kDa fragment of FLNA in C-terminal (C-FLNA) in Sema3d-interfered HCC cells. The Western blot showed that knockdown of Sema3d in PLC/PRF/5 upregulated full-length FLNA and phosphorylated FLNA and downregulated nuclear C-FLNA. In the contrast, overexpression of Sema3d in HCCLM3 obtained an opposite result (**Figure 5D**). Moreover, the staining of C-FLNA by IF revealed that Sema3d upregulated C-FLNA and promoted the nucleus translocation (**Figure 5E**). Summarily, these results suggest that Sema3d directly combines with FLNA and prevents its phosphorylation, allowing FLNA to be cleaved to the 90-kDa fragment and translocated to the nucleus, which could be the mechanism by which low Sema3d promotes the progression of HCC cells.

### Downregulation of Sema3d Promotes EMT in HCC

**Figure 6:**
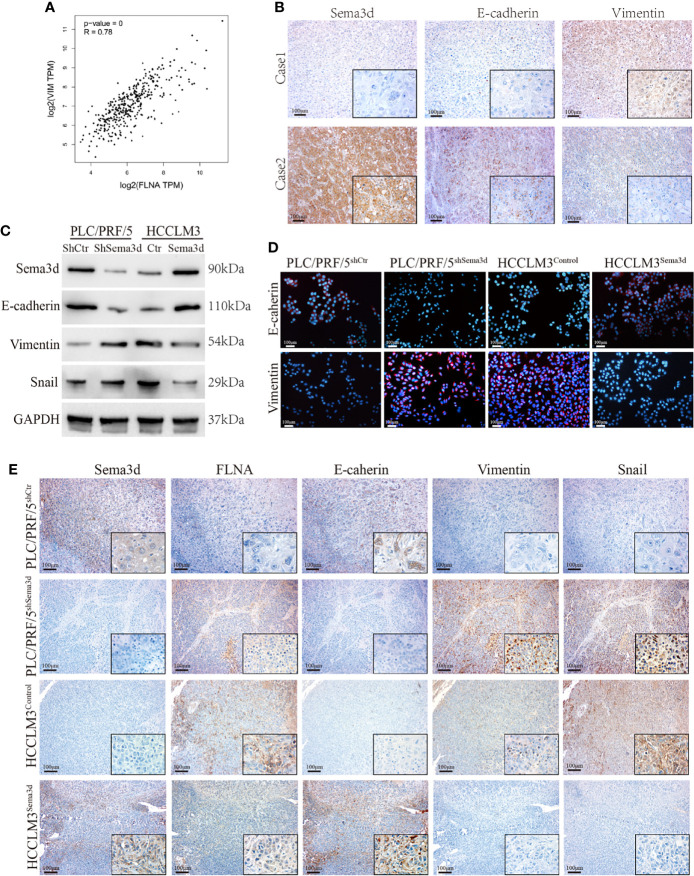
Downregulation of Sema3d promotes EMT in HCC. **(A)** Correlation of FLNA and vimentin mRNA expression levels was analyzed from TCGA. **(B)** Representative image of Sema3d, E-cadherin, and vimentin expression level in HCC tissue detected by IHC. **(C)** EMT marker proteins in PLC/PRF/5^shSema3d^ and HCCLM3^Sema3d^ cells and their corresponding control cells were detected by Western blot. **(D)** EMT marker proteins in Sema3d-interfered PLC/PRF/5 and HCCLM3 cells detected by IF. **(E)** IHC was performed to detect the FLNA and EMT marker proteins in consecutive sections of a liver orthotopic xenograft tumor derived from Sema3d-interfered PLC/PRF/5 and HCCLM3 cells.

In previous results, we found that Sema3d remodeled the cytoskeleton and changed cellular morphology *via* interaction with FLNA. Moreover, the result of GSEA from TCGA datasets also indicates that Sema3d might regulate EMT (**Supplementary Figure S7**). In addition, we found that FLNA had a high correlation with Vimentin from data from TCGA (**Figure 6A**), another cytoskeleton protein known as a mesenchymal marker. These results all indicated that Sema3d might affect EMT in HCC. Therefore, we examined EMT markers E-cadherin and vimentin in Sema3d low- and high-expressed HCC tissue by IHC (**Figure 6B**), and the Spearman’s rank correlation found that Sema3d expression level positively correlates with E-cadherin and negatively related with Vimentin (**Supplementary Figure S8**). We then detected EMT marker proteins in Sema3d interfered with PLC/PRF/5 and HCCLM3 cells by Western blot and IF (**Figures 6C, D**) and revealed that knockdown of Sema3d in PLC/PRF/5 downregulated epithelial marker E-cadherin and upregulated mesenchymal marker vimentin, and overexpression of Sema3d in HCCLM3 obtained the opposite result. Finally, we detected FLNA and EMT markers in consecutive sections of liver orthotopic xenograft tumors derived from Sema3d-interfered PLC/PRF/5 and HCCLM3 cells, which indicated that FLNA, Vimentin, and Snail decreased and E-cadherin increased in Sema3d overexpressed tumors compared with the controls, and the opposite results observed in Sema3d knockdown tumors (**Figure 6E**). Together, these results indicated that downregulation of Sema3d promoted EMT *via* FLNA in HCC.

### Sema3d Suppressed Pi3k/AKT Signaling and Restrained HCC Proliferation, Metastasis, and EMT Through FLNA

**Figure 7:**
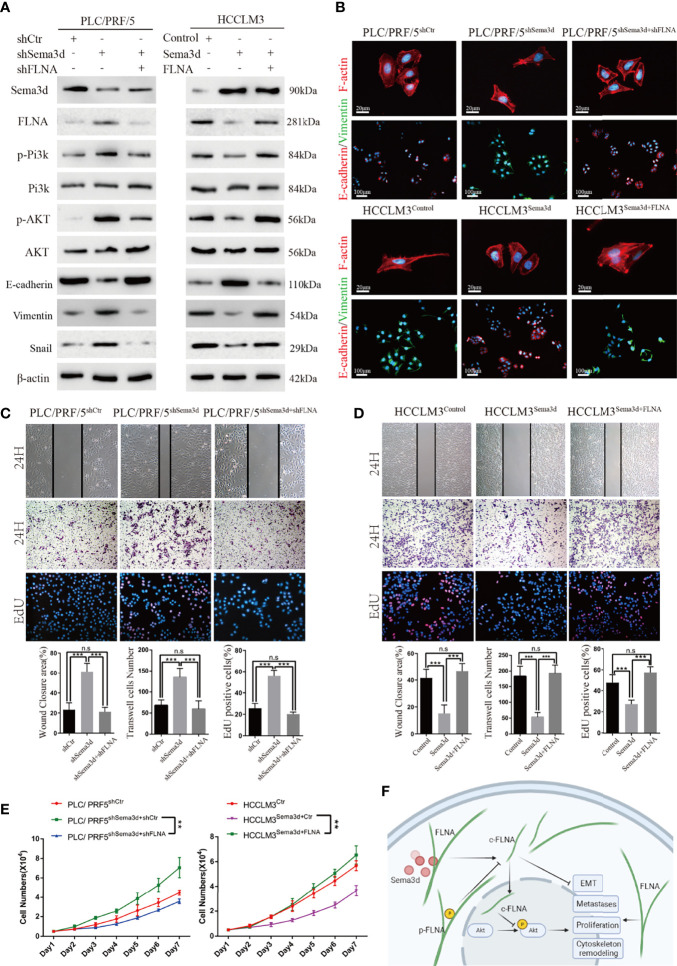
Sema3d suppressed Pi3k/AKT signaling and restrained HCC proliferation, metastasis, and EMT through FLNA. **(A)** Protein expression level was analyzed by Western blot and showed that Sema3d regulated the phosphorylation level of Pi3k and AKT and expression of EMT markers through FLNA. **(B)** F-actin and EMT marker proteins in Seme3d- and FLNA-interfered HCC cells were detected by IF. **(C)** Wound healing, Transwell invasion, and EdU staining assays detected the migration, invasion, and proliferation of HCCLM3^Sema3d^ ectopic expression of FLNA. **(D)** Wound healing, Transwell invasion, and EdU staining assays detected the migration, invasion, and proliferation of PLC/PRF/5^shSema3d^ knockdown FLNA. **(E)** MTT assay detected a proliferation of HCCLM3^Sema3d^ ectopic expression of FLNA and PLC/PRF/5^shSema3d^ interference of FLNA compared with the corresponding control cells. **(F)** Sema3d restrained hepatocellular carcinoma proliferation, metastases, EMT, and cytoskeleton remodeling through inactivating Pi3k/Akt *via* interaction with FLNA. ^**^ *p* < 0.01; ^***^ *p* < 0.001. n.s, no significance.

It has been proven that Sema3d suppresses Pi3k/Akt signaling in HCC and interacts with FLNA. However, whether Sema3d regulates Pi3k/Akt signaling *via* FLNA in HCC is unknown. To further test whether Pi3k/Akt signaling was regulated by FLNA, the FLNA-shRNA was transfected into PLC/PRF5^shSema3d^ cells, and an FLNA ectopic expression plasmid was transfected into HCCLM3^Sema3d^ cells. Ectopic expression of FLNA in HCCLM3^Sema3d^ cells restored the phosphorylation level of Pi3k, Akt, Vimentin, and Snail, which was decreased by Sema3d overexpression as shown earlier. Knockdown of FLNA in PLC/PRF5^shSema3d^ cells got the opposite result (**Figure 7A**). We also detected F-actin and EMT markers in FLNA interfered with PLC/PRF5^shSema3d^ cells by IF (**Figure 7B**), revealing that knockdown of FLNA in PLC/PRF5^shSema3d^ cells induced E-cadherin expression and decreased Vimentin expression and presented a cobblestone-like morphology with shrinkable F-actin fibers. In contrast, ectopic expression of FLNA in HCCLM3^Sema3d^ cells had the inverse effect. The wound-healing, Transwell, EdU staining, and MTT assays indicated that knockdown of FLNA in PLC/PRF5^shSema3d^ cells decreased and ectopic expression of FLNA in HCCLM3^Sema3d^ cells increased the migration, invasion, and proliferation capacity (**Figures 7C–E**). These results indicated that Sema3d restrained Pi3k/AKT signaling and suppressed HCC proliferation, metastasis, EMT, and remodeled the cytoskeleton through FLNA.

## Discussion

HCC is the sixth most commonly diagnosed cancer and the fourth leading cause of cancer-related death worldwide (1, 33). Comprehensive treatment combined with surgery, locoregional therapies, targeted therapy, and immune therapy has gradually replaced surgery alone, but the prognosis still remains unsatisfactory (34). Precision medicine highlights that diseases should be classified into subtypes depending on the degree of heterogeneity and clinical characteristics, then develop precise treatment plans for disease subtypes to obtain the best overall outcomes (35, 36). The codevelopment of predictive biomarkers together with novel targeted therapies is essential to overcome this issue (37). In this case, there is an urgent need to discover more accurate molecular biomarkers and new therapeutic targets.

Notably, plenty of evidence demonstrated that selected members of the semaphorin family may represent useful biomarkers for the prognostic evaluation of various human tumors. Moreover, some preclinical research supports some of these molecules as relevant targets for cancer therapy (38). For instance, a humanized version of this anti-Sema4D antibody has been validated for the treatment of solid tumors in clinical trials (39). In our study, for the first time, we show that Sema3d, one of the semaphorins, is capable of suppressing HCC proliferation, invasion, and metastasis through Pi3k/AKT signaling and also has the potential to be an effective biomarker to predict the prognosis of HCC. The previous study has demonstrated that the primary receptors for semaphorins are plexins and neuropilins (40). Sema3d can act as a repellent signal to axons in zebrafish (41), inhibit the migration capacity and mediates cytoskeletal reorganization of human endothelial cells dependent or independent of these receptors (17). Increasing evidence has shown that semaphorins can also affect signal transduction through a nonplexin or nonneurropilin receptor complex (18, 19, 42). Actually, Sema3d has been proven to affect multisignaling pathways in various cells. For instance, Sema3d inhibits parathyroid cell proliferation by decreasing the EGFR/ErbB signaling pathway (19); Sema3d also inhibits tumor development following implantation in the cortex of mouse brains and significantly prolongs the survival of these mice (18), a function in cancer consistent with our research. Whatever the case, Sema3d and PlxnD1 have been shown to promote metastasis in various types of cancer and regulate the EMT of pancreatic ductal adenocarcinoma (43), which reveals that the complex function of Sema3d, and perhaps play the opposite role in a variety of cancers or cells through different receptors or signaling pathways.

Sema3d has multifunctions and causes the cellular response to depend on the receptor complexes or the downstream targets, and different receptors can produce opposing responses to the same ligand (16). In our research, we conducted RNA sequencing of Sema3d overexpressed HCCLM3 cells and combined GSEA in TCGA dataset. The results showed that Pi3k/AKT signaling was downregulated in Sema3d overexpressed HCCLM3 cells and highly expressed cases, which indicated that Sema3d regulated HCC through Pi3k/AKT signaling. The hypothesis was then proved in the cells interfered with by the Pi3k/AKT signaling inhibitor. A myriad of reports disclosed that the Pi3k/Akt signaling pathway participates in various processes of human cancers such as cell proliferation, migration, angiogenesis, and lymphangiogenesis (44). For example, lncRNA HOXB-AS3 and LncRNA TCL6 exacerbate the proliferative and migratory abilities of lung cancer cells with the activation of the PI3K/AKT pathway (45, 46). Consistent with previous studies, our study also confirmed the important role of Sema3d *via* in affecting Pi3k/Akt signaling in HCC.

Previously, FLNA has been proven to regulate cytoskeleton structure and cell migration in organogenesis (47) and is also involved in multiple cellular pathways. It can be linked as an important factor in many cancer-promoting steps (48). FLNA’s function in the cytoplasm as a scaffolding protein and its vital importance in cell adhesion and migration can transform it into an extremely potent cancer-promoting protein (49). FLNA’s new role in the nucleus has led researchers to evaluate the differences that arise from the localization of the protein (32, 50). Cytoplasmic FLNA is often highly overexpressed in metastatic cancers, including breast and prostate. Specifically, in metastatic prostate cancer, cytoplasmic FLNA is phosphorylated at S2152, which prevents its cleavage at the hinge region to the 90-kDa fragment, whereas in less aggressive or benign prostate tumors, the 90-kDa FLNA is found in the nucleus. Some studies have suggested a correlation between FLNA and Pi3k/Akt pathway in prostate and colorectal cancer (51). In line with the former study, our research firstly revealed this relationship and FLNA play an important role in HCC.

In our research, Sema3d also exhibited an important function in cytoskeleton modeling and EMT. As known, EMT plays a crucial role in the early steps of HCC metastasis when cells lose cell–cell contacts due to ablation of E-cadherin and acquire increased motility to spread into surrounding or distant tissues (52). We also demonstrated the role of EMT in HCC progression in early studies (10, 53). More than that, Sema3d was demonstrated to regulate EC morphology and actin network organization *via* neuropilin1 (17), which were also verified in HCC by our research but through a totally different mechanism. In our view, the roles of FLNA, the cytoskeleton actin-binding protein, and scaffolding protein, possess more powerful regulation in cytoskeleton remodeling. It is also worth pointing out that the actin cytoskeleton plays an important role in the maintenance of cell shape, cell movement, and transportation of substances in cells and mediates cell response to cytokinesis (11, 18). In cancer cells, the actin cytoskeleton regulates migration and tumor morphogenesis and even plays a pivotal role in driving breast cancer cell resistance to natural killer cells (11, 19).

In conclusion, we proved that downregulated Sema3d in HCC significantly correlated with poor prognosis. Low Sema3d expression has the potential to serve as an independent risk-predicting marker for HCC patients. Sema3d restrained the progression of hepatocellular carcinoma proliferation, invasion, and metastasis through inactivating Pi3k/Akt, which may serve as a novel prognostic predictor and a potential therapeutic target for HCC patients.

## Data Availability Statement

The datasets presented in this study can be found in online repositories. The names of the repository/repositories and accession number(s) can be found in the article/**Supplementary Material**.

## Ethics Statement

The studies involving human participants were reviewed and approved by the Ethics Committee of Xiangya Hospital, Central South University. The patients/participants provided their written informed consent to participate in this study. The animal study was reviewed and approved by the Ethics Committee of Xiangya Hospital, Central South University.

## Author Contributions

YL and LY conceived the study and wrote the manuscript. YL and BS conducted the experiments and contributed to the data analysis. YL, BS, CX, FZ, MC, and LY collected clinical samples and corresponding clinical data. YL and LY revised the manuscript. All authors read and approved the final manuscript.

## Funding

This work was supported by the National Natural Science Foundation of China (81773139), the Key Project of the National Natural Science Foundation of China (81330057), and Specialized Research Fund for Doctoral Program of Higher Education of China (20130162130007).

## Conflict of Interest

The authors declare that the research was conducted in the absence of any commercial or financial relationships that could be construed as a potential conflict of interest.

## Publisher’s Note

All claims expressed in this article are solely those of the authors and do not necessarily represent those of their affiliated organizations, or those of the publisher, the editors and the reviewers. Any product that may be evaluated in this article, or claim that may be made by its manufacturer, is not guaranteed or endorsed by the publisher.

## Abbreviations

HCC, hepatocellular carcinoma; Sema3d, semaphorin 3d; GSEA, gene set enrichment analysis; co-IP, coimmunoprecipitation; LC-MS, liquid chromatography-tandem mass spectrometry; EMT, epithelial–mesenchymal transition; ANLTs, adjacent nontumoral liver tissues; PHHs, primary human hepatocytes; SLHCC, solitary large hepatocellular carcinoma; NHCC, nodular hepatocellular carcinoma; SHCC, small hepatocellular carcinoma; IHC, immunohistochemistry; IF, immunofluorescence; TCGA, The Cancer Genome Atlas; FLNA, filamin-A; C-FLNA, 90 kDa fragment FLNA in C terminal.
