# Supplementary material for: Sema3d Restrained Hepatocellular Carcinoma Progression Through Inactivating Pi3k/Akt Signaling via Interaction With FLNA
Source: Front Oncol. 2022 Jul 25;12:913498. doi: 10.3389/fonc.2022.913498 (PMC9358705; doi:10.3389/fonc.2022.913498)
Supplement: Supplementary file 1 [file DataSheet_1.pdf]

Fig. S1 Kaplan-Meier curves of the TCGA-LIHC data.

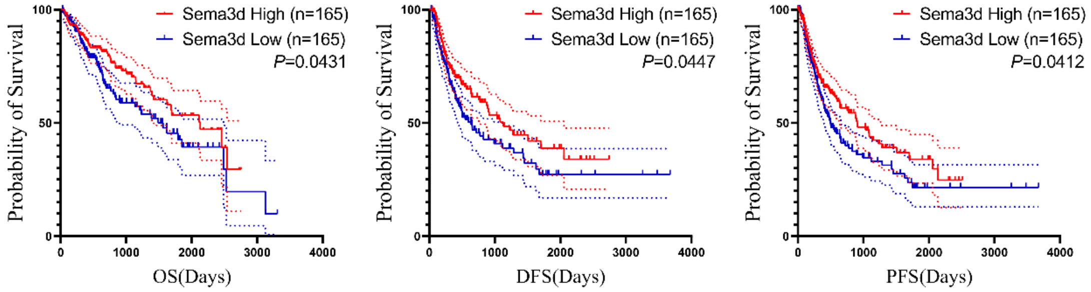

Fig. S1 Kaplan-Meier curves of the overall survival (OS), disease free survival (DFS), and progression free survival (PFS) in TCGA, the result indicated that low Sema3d expression associated with worse OS, DFS and PFS.

Fig. S2 Testing the effect of ectopic expression in HCCLM3 and knock down in PLC/PRF/5.

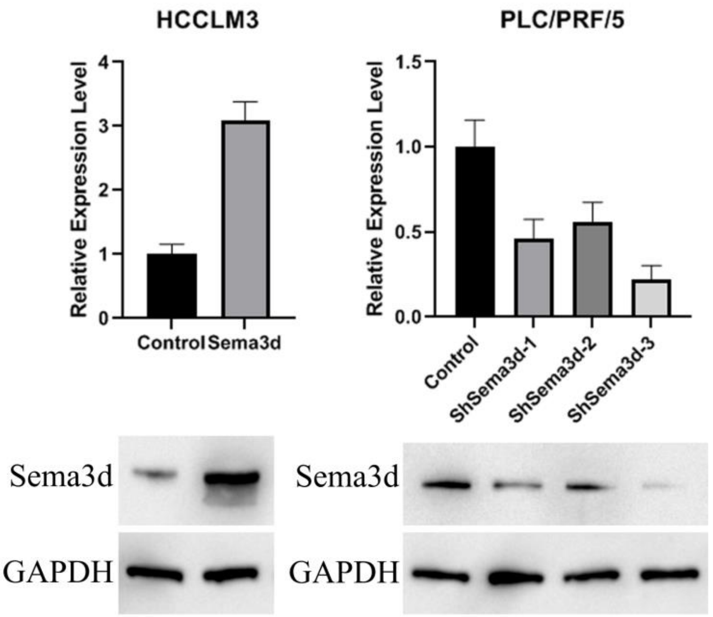

Fig. S2 Testing the effect of ectopic expression in HCCLM3 and knock down in PLC/PRF/5. Sema3d mRNA and protein expression levels were detected in Sema3d ectopic expression and knock down HCC cells. PLC/PRF/5-ShSema3d-3 was the most effective knockdown among the sequences and was chosen for further study.

Fig. S3 EdU assay indicated that Sema3d inhibited proliferation of HCC cells

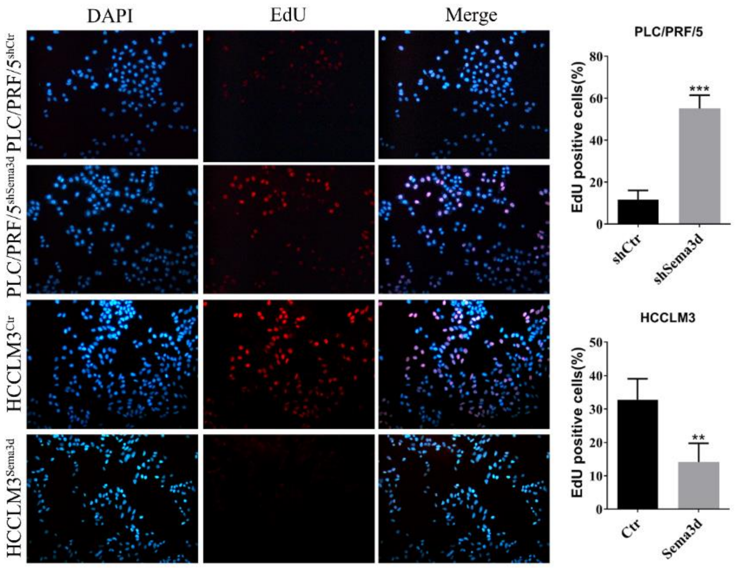

Fig. S3 EdU assay indicated that Sema3d inhibited proliferation of HCCLM3^{Sema3d} and PLC/PRF/5^{shCtr} cells compared with the corresponding cells.

Fig. S4 H&E staining of liver orthotopic xenograft tumors

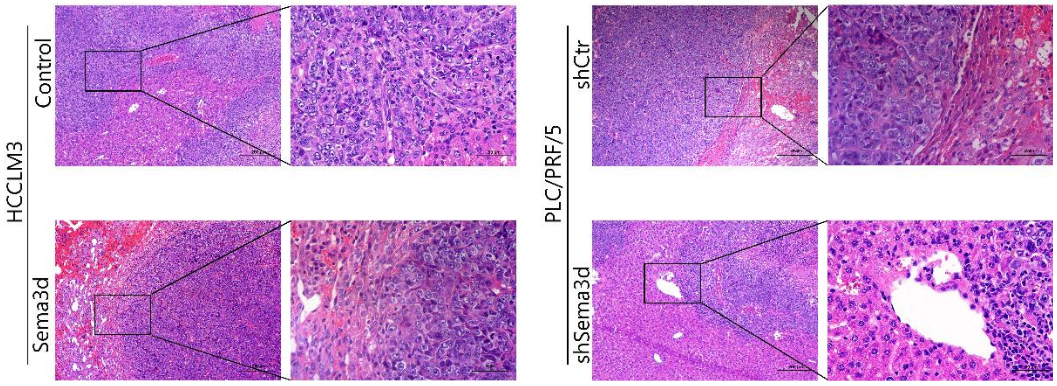

Fig. S4. H&E staining of liver orthotopic xenograft tumors revealed that the PLC/PRF/5^{shSema3d} and HCCLM3^{Ctr} cells derived tumors exhibit character of invasive extension with irregular border and multi nodular, but the PLC/PRF/5^{shCtr} and HCCLM3^{Sema3d} derived tumors demonstrated expansive growth with well-defined borders.

Fig. S5 RNA-sequencing of the HCCLM3^{Sema3d} and HCCLM3^{NC} cells.

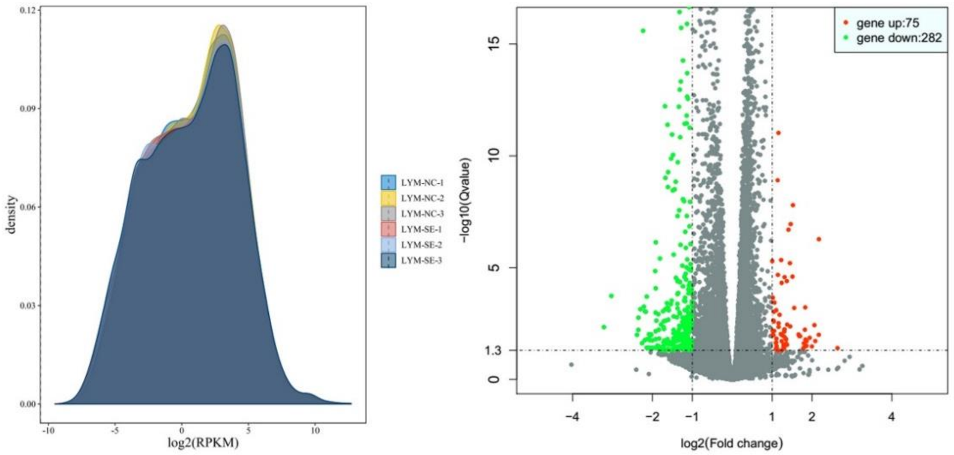

Fig.S5 RNA-sequencing of the HCCLM3^{Sema3d} and HCCLM3^{NC} cells. RPKM(Reads Per Kb per Million reads) was used to eliminate effect of the differences in sequencing volume. The Volcano Plots indicates that Sema3d ectopic expression in HCCLM3 decreased expression of 282 genes and increased 75 genes.

Fig. S6 Heat map of Pi3k/Akt signaling related genes downregulated in HCCLM3^{Sema3d} cells in RNA-sequencing.

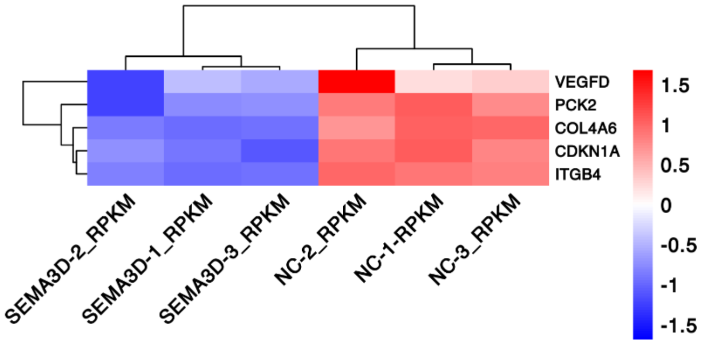

Fig.S6 RNA-sequencing of the HCCLM3^{Sema3d} and HCCLM3^{NC} cells revealed several Pi3k/Akt signaling related genes were downregulated by Sema3d, which indicated that Sema3d might inhibited progression of HCC cells via inactivating Pi3k/Akt signaling.

Fig. S7 Pi3k-AKT-mTor signaling and EMT significantly enriched in Sema3d-Low group.

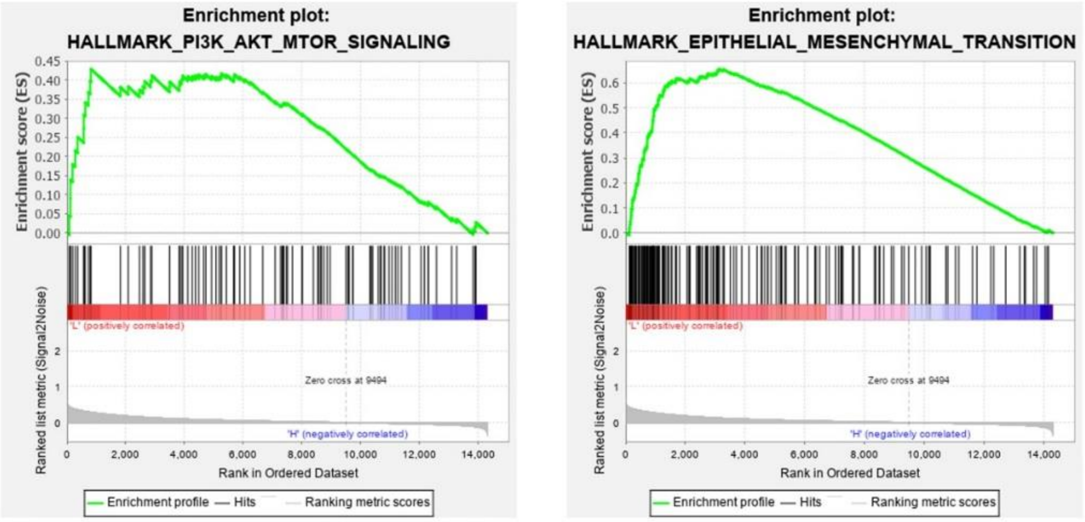

Normalized Enrichment Score (NES)=1.7089646
Nominal p-value=0.010288066

Normalized Enrichment Score (NES)=1.5342363
Nominal p-value =0.022222223

Fig. S7 GSEA was performed to analysis Hallmark gene sets in TCGA cohort, Pi3k-AKT-mTor signaling ranks the top one pathway and EMT ranks the fourth pathway enriched in Sema3d-Low expression group.

Fig. S8 Correlation between expression of Sem3d and EMT marker in HCC

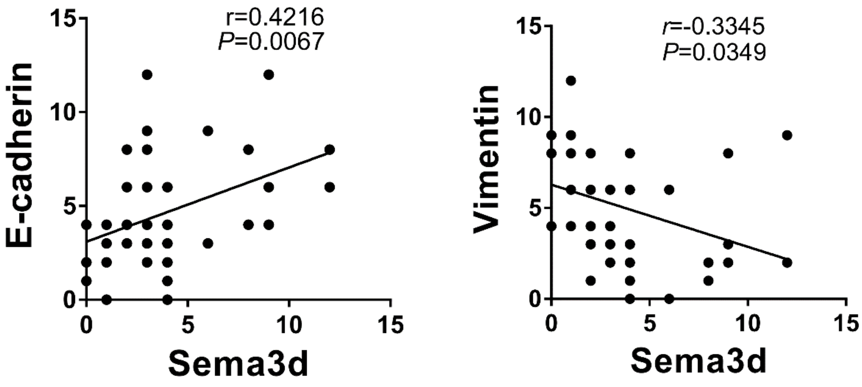

Fig. S8 Spearman rank correlation of the IHC score found that Sema3d expression level positively correlates with E-cadherin and negatively related with Vimentin.

Fig. S9 Invasion capacity of 8 HCC cell lines

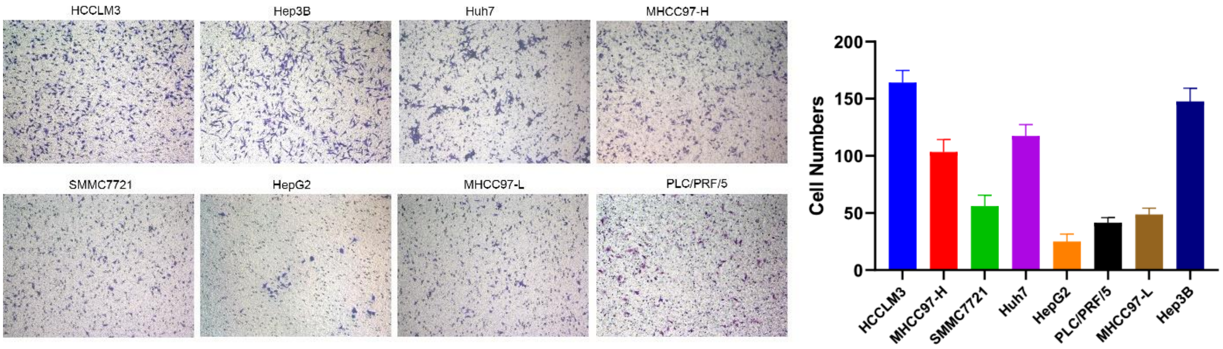

Transwell invasion assay of the 8 HCC cell lines (HCCLM3, Hep3B, Huh7, MHCC97-H, SMMC7721, HepG2, MHCC97-L and PLC/PRF/5) in the same number of cells and without any interfered. After 24 hours, the result indicates that invasion capacity of HCCLM3, Hep3B, Huh7, MHCC97-H significantly stronger than the SMMC7721, HepG2, MHCC97-L and PLC/PRF/5, the mRNA expression level mainly consistent with the degree of cell invasion capacity of HCC cell lines.

Fig. S10 Proliferation vitality of Sema3d overexpressed PHH, PLC/PRF/5 and HCCLM3 cells and the corresponding control cells.

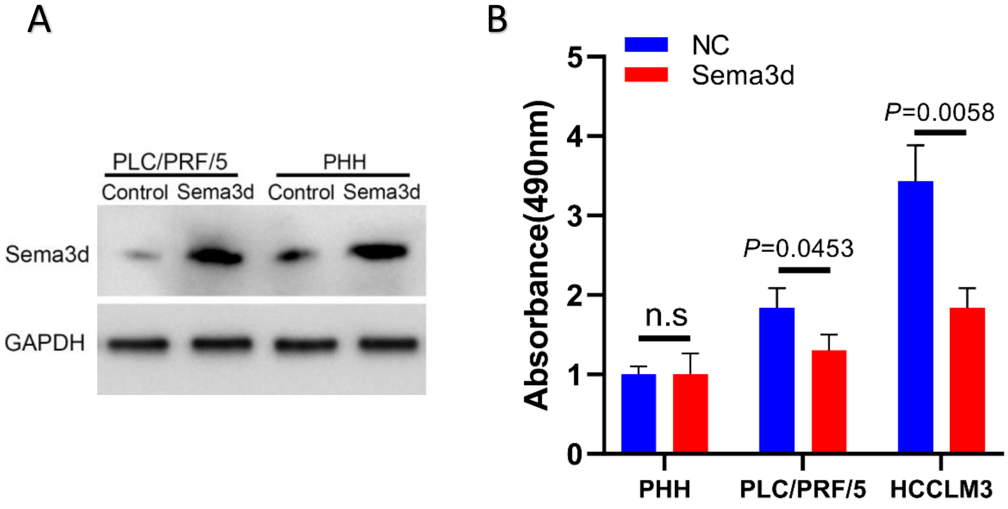

Western blot was performed to examine the effect of Sema3d ectopic expression in PHH and PLC/PRF/5 cells (Fig. S10 A). MTT assay was performed to test the proliferation vitality of PHH, PLC/PRF/5 and HCCLM3 cells transfected Sema3d ectopic expression lentivirus or control lentivirus, and the absorbance (490nm) was tested at the 5th day (Fig. S10 B). The proliferation vitality was not significantly affected in PHH cells, but significantly decreased in PLC/PRF/5 and HCCLM3 cell.
