# Supplementary material for: Sema3d Restrained Hepatocellular Carcinoma Progression Through Inactivating Pi3k/Akt Signaling via Interaction With FLNA
Source: Front Oncol. 2022 Jul 25;12:913498. doi: 10.3389/fonc.2022.913498 (PMC9358705; doi:10.3389/fonc.2022.913498)
Supplement: Supplementary file 2 [file DataSheet_2.pdf]

Supplementary Table S1. Correlation between Sema3d expression and clinicopathologic characteristics of HCC patients in training cohort and validation cohort.

Clinicopathologic variables	Training cohort				Validation cohort			
		Sema3d				Sema3d expression		
	n	High	Low	*P*	n	High	Low	*P*
**Gender**		69	111	0.132		55	65	0.236
Female	39	19	20		23	8	15	
Male	141	50	91		97	47	50	
**Age (years)**				0.147				0.112
≤50	116	49	67		87	36	51	
>50	64	20	44		33	19	14	
**AFP(**ng/ml**)**				0.229				0.364
<20	29	14	15		22	12	10	
≥20	151	55	96		98	43	55	
**Hepatitis B status**				0.105				0.249
Negative	29	15	14		27	15	12	
Positive	151	54	97		93	40	53	
**Liver cirrhosis**				0.152				0.299
Absent	64	29	35		48	25	23	
Present	116	40	76		72	31	42	
**Child-Pugh classification**				0.293				0.184
A	113	40	73		73	37	36	
B	67	29	38		47	18	29	
**Tumor size(cm)**				**0.032**				**0.003**
≤ 5	74	35	38		46	29	17	
> 5	106	34	72		74	26	48	
**Tumor nodule number**				**0.034**				**0.002**
Solitary	113	50	63		69	40	29	
Multiple (≥ 2)	67	19	48		51	15	36	
**Capsulation formation**				0.207				0.129
Presence	91	39	52		50	27	23	
Absence	89	30	59		70	28	42	
**Edmondson-Steiner grade**				0.059				0.225
Ⅰ&Ⅱ	78	36	42		53	21	32	
Ⅲ&Ⅳ	102	33	69		67	34	33	
**Microvascular invasion**				**0.013**				**0.005**
Absence	121	54	67		71	40	31	
Presence	59	15	44		49	15	34	
**Macrovascular invasion**				**0.032**				**0.029**
Absence	143	61	82		102	51	51	
Presence	37	8	29		18	4	14	
**BCLC stage**				**0.002**				**0.001**
0&A	83	42	41		51	32	19	
B&C	97	27	70		69	23	46	
**TNM stage**				**0.012**				**0.013**
Ⅰ	73	36	37		53	31	22	
Ⅱ&Ⅲ	107	33	74		67	24	43	

**CNLC stage**				**<0.001**				**0.009**
Ⅰ	86	45	41		48	29	19	
Ⅱ&Ⅲ	94	24	70		72	26	46	

Abbreviations: AFP, alpha-fetoprotein; TNM, tumor node metastasis; BCLC, Barcelona Clinic Liver Cancer; CNLC, China Clinic Liver Cancer.

Supplementary Table S2. Univariate and multivariate analyses of risk factors associated with overall survival and disease-free survival of HCC patients in training cohort.

Clinicopatholo-gic variables	OS		DFS	
	Univariate Analysis	Multivariate Analysis	Univariate Analysis	Multivariate Analysis
	P HR(95% CI)	***P*** HR(95% CI)	***P*** HR(95% CI)	***P*** HR(95% CI)
**Gender**	0.940	NA	0.271	NA
Female	1		1	
Male	1.017(0.661-1.565)		1.224(0.854-1.754)	
**Age (years)**	0.389	NA	0.100	NA
≤50	1		1	
>50	1.183(0.807-1.733)		1.304(0.951-1.790)	
**AFP(**ng/ml**)**	**0.033**	0.077	**<0.001**	0.057
<20	**1**	1	**1**	1
≥20	**1.600(1.039-2.464)**	1.573(0.953-2.597)	**1.993(1.417-2.804)**	1.487(0.989-2.238)
**Hepatitis B status**	0.606	NA	0.152	NA
Negative	1		**1**	
Positive	1.116(0.736-1.692)		**1.292(0.910-1.835)**	
**Liver cirrhosis**	0.138	NA	0.064	NA
Absent	1		1	
Present	1.345(0.909-1.989)		1.351(0.983-1.858)	
**Child-Pugh classification**	0.145	NA	0.104	NA
A	1		1	
B	1.318(0.909-1.909)		1.320(0.945-1.845)	
**Tumor size (cm)**	**0.002**	0.337	**0.013**	0.529
≤ 5	**1**	1	**1**	1
> 5	**1.774(1.228-2.563)**	1.236(0.802-1.907)	**1.514(1.091-2.100)**	1.127(0.777-1.634)
**Tumor nodule number**	**<0.001**	0.052	**0.018**	0.063
Solitary	**1**	1	**1**	1

Multiple (≥ 2)	**2.673(1.840-3.883)**	1.500(0.996-2.413)	**1.476(1.068-2.038)**	1.472(0.979-2.212)
**Capsulation formation**	**0.039**	0.149	**0.009**	0.066
Presence	**1**	1	**1**	1
Absence	**1.472(1.020-2.124)**	1.388(0.674-1.708)	**1.536(1.113-2.121)**	1.392(0.978-1.981)
**Edmondson-Steiner grade**	**0.008**	0.140	**0.007**	0.190
Ⅰ&Ⅱ	**1**	1	**1**	1
Ⅲ&Ⅳ	**1.643(1.139-2.371)**	1.364(0.903-2.059)	**1.571(1.134-2.177)**	1.297(0.879-1.915)
**Microvascular invasion**	**0.006**	**0.005**	**0.003**	**0.017**
Absence	**1**	**1**	**1**	**1**
Presence	**1.707(1.169-2.492)**	**1.996(1.231-3.236)**	**1.686(1.199-2.370)**	**1.541(1.080-2.197)**
**Macrovascular invasion**	**<0.001**	**0.002**	**<0.001**	**<0.001**
Absence	**1**	**1**	**1**	**1**
Presence	**2.206(1.362-3.012)**	**1.971(1.289-3.013)**	**2.047(1.394-3.003)**	**2.078(1.399-3.086)**
**BCLC stage**	**0.018**	**0.025**	**0.003**	**0.034**
0&A	**1**	**1**	**1**	**1**
B&C	**1.628(1.086-2.441)**	**1.731(1.071-2.797)**	**1.665(1.191-2.328)**	**1.531(1.033-2.268)**
**TNM stage**	**0.014**	**0.017**	**0.001**	**<0.001**
Ⅰ	**1**	**1**	**1**	**1**
Ⅱ&Ⅲ	**1.635(1.105-2.421)**	**1.783(1.107-2.873)**	**1.821(1.294-2.563)**	**2.124(1.476-3.076)**
**CNLC stage**	**<0.001**	**0.007**	**0.003**	**0.004**
Ⅰ	**1**	**1**	**1**	**1**
Ⅱ&Ⅲ	**1.971(1.366-2.844)**	**1.777(1.168-2.702)**	**1.616(1.171-2.230)**	**1.801(1.213-2.673)**
**Sema3d expression**	**0.013**	**0.019**	**0.001**	**0.022**
Low	**1**	**1**	**1**	**1**
High	**1.579(1.103-2.262)**	**1.502(1.069-2.110)**	**1.840(1.276-2.653)**	**1.484(1.058-2.081)**

Abbreviations: AFP, alpha-fetoprotein; HBsAg, hepatitis B surface antigen; TNM, tumor node metastasis; BCLC, Barcelona Clinic Liver Cancer; CNLC, China Clinic Liver Cancer.

Supplementary Table S3. Univariate and multivariate analyses of risk factors associated with overall survival and disease-free survival of HCC patients in validation cohort.

**Clinicopatholo-gic variables**	**OS**		**DFS**	
	**Univariate Analysis**	**Multivariate Analysis**	**Univariate Analysis**	**Multivariate Analysis**
	P **HR(95% CI)**	***P*** **HR(95% CI)**	***P*** **HR(95% CI)**	***P*** **HR(95% CI)**
**Gender**	0.489	NA	0.496	NA
Female	1		1	
Male	1.213(0.703-2.093)		1.195(0.715-1.998)	
**Age (years)**	0.510	NA	0.889	NA
≤50	1		1	
>50	1.173(0.729-1.887)		1.033(0.654-1.633)	
**AFP(**ng/ml**)**	**0.039**	0.317	**0.013**	0.132
<20	**1**	1	**1**	1
≥20	**1.702(1.028-2.818)**	1.320(0.766-2.277)	**1.819(1.135-2.914)**	1.478(0.889-2.458)
**Hepatitis B status**	0.215	NA	0.175	NA
Negative	1		1	
Positive	1.413(0.818-2.440)		1.418(0.857-2.347)	
**Liver cirrhosis**	0.131	NA	0.142	NA
Absent	1		1	
Present	1.417(0.902-2.227)		1.368(0.900-2.078)	
**Child-Pugh classification**	0.162	NA	0.053	NA
A	1		1	
B	1.365(0.883-2.109)		1.497(0.994-2.252)	
**Tumor size (cm)**	0.064	NA	0.233	NA
≤ 5	1		1	
> 5	1.545(0.976-2.448)		1.289(0.849-1.957)	
**Tumor nodule number**	**0.017**	**0.002**	**0.001**	**0.031**
Solitary	**1**	**1**	**1**	**1**

Multiple (≥ 2)	**1.751(1.105-2.776)**	**2.242(1.358-3.701)**	**1.995(1.327-3.000)**	**1.643(1.048-2.578)**
**Capsulation formation**	**0.023**	0.371	**0.032**	0.243
Presence	**1**	1	**1**	1
Absence	**1.682(1.074-2.636)**	1.362(0.692-2.678)	**1.576(1.040-2.388)**	1.460(0.773-2.757)
**Edmondson-Steiner grade**	**0.019**	**0.028**	**0.007**	0.011
Ⅰ&Ⅱ	**1**	**1**	**1**	1
Ⅲ&Ⅳ	**1.692(1.091-2.625)**	**1.691(1.057-2.705)**	**1.750(1.161-2.638)**	1.802(1.145-2.835)
**Microvascular invasion**	**0.008**	**0.029**	**0.005**	**0.012**
Absence	**1**	**1**	**1**	**1**
Presence	**1.801(1.168-2.775)**	**1.713(1.058-2.773)**	**1.778(1.187-2.665)**	**1.870(1.150-3.042)**
**Macrovascular invasion**	**0.006**	**0.002**	**0.002**	**0.027**
Absence	**1**	**1**	**1**	**1**
Presence	**1.944(1.212-3.118)**	**2.242(1.358-3.701)**	**2.049(1.300-3.231)**	**1.795(1.067-3.019)**
**BCLC stage**	**0.040**	**0.038**	**0.019**	**0.009**
0&A	**1**	**1**	**1**	**1**
B&C	**1.774(1.028-3.064)**	**1.850(1.035-3.308)**	**1.832(1.105-3.034)**	**2.406(1.192-3.513)-**
**TNM stage**	**0.002**	**0.018**	**<0.001**	**0.013**
Ⅰ	**1**	**1**	**1**	**1**
Ⅱ&Ⅲ	**2.078(1.320-3.272)**	**1.913(1.120-3.269)**	**2.154(1.409-3.292)**	**1.880(1.141-3.097)**
**CNLC stage**	**0.012**	**0.019**	**0.006**	**0.026**
Ⅰ	**1**	**1**	**1**	**1**
Ⅱ&Ⅲ	**1.807(1.140-2.865)**	**1.782(1.099-2.890)**	**1.828(1.191-2.805)**	**1.842(1.077-3.152)**
**Sema3d expression**	**0.002**	**0.031**	**0.004**	**0.021**
High	**1**	**1**	**1**	**1**
Low	**1.865 (1.260-2.761)**	**1.567(1.043-2.355)**	**1.788(1.207-2.647)**	**1.486(1.061-2.083)**

Abbreviations: AFP, alpha-fetoprotein; HBsAg, hepatitis B surface antigen; TNM, tumor node metastasis; BCLC, Barcelona Clinic Liver Cancer; CNLC, China Clinic Liver Cancer.

Supplementary Table S4. Clinicopathologcal characteristics of HCC patients in training cohort and validation cohort.

Clinicopathologic variables	Counts		*P*
	Training cohort	Validation cohort	
**Gender**			0.600
Female	39(21.67%)	23(19.17%)	
Male	141(78.33%)	97(80.83%)	
**Age (years)**			0.144
≤60	116(64.44%)	87(72.50%)	
>60	64(35.56%)	33(27.50%)	
**AFP(**ng/ml**)**			0.804
<20	31(17.22%)	22(18.33%)	
≥20	149(82.78%)	98(81.67%)	
**Hepatitis B status**			0.615
Negative	29(16.11%)	27(18.33%)	
Positive	151(83.89%)	93(81.67%)	
**Liver cirrhosis**			0.436
Absent	64(35.56%)	48(40.00%)	
Present	116(64.44%)	72(60.00%)	
**Child-Pugh classification**			0.734
A	113(62.78%)	73(60.83%)	
B	67(37.22%)	47(39.17%)	
**Tumor size (cm)**			0.630
≤ 5	74(41.11%)	46(38.33%)	
> 5	106(58.89%)	74(61.67%)	
**Tumor nodule number**			0.359
Solitary	113(62.78%)	69(57.50%)	
Multiple (≥ 2)	67(37.22%)	51(42.50%)	
**Capsulation formation**			0.131
Presence	91(50.56%)	50(41.67%)	

Absence	89(49.44%)	70(58.33%)	
**Edmondson-Steiner grade**			0.886
Ⅰ&Ⅱ	78(43.33%)	53(44.17%)	
Ⅲ&Ⅳ	102 (56.67%)	67(55.83%)	
**Microvascular invasion**			0.154
Absence	121(67.22%)	71(59.17%)	
Presence	59(32.78%)	49 (40.83%)	
**Macrovascular invasion**			0.223
Absence	143(79.44%)	102(85.00%)	
Presence	37(20.56%)	18(15.00%)	
**BCLC stage**			0.538
0&A	83(46.11%)	51(42.50%)	
B&C	97 (53.89%)	69(65.38%)	
**TNM stage**			0.535
Ⅰ	73(40.56%)	53(44.17%)	
Ⅱ&Ⅲ	107(59.44%)	67(55.83%)	
**CNLC stage**			0.184
Ⅰ	86(44.78%)	48(40.00%)	
Ⅱ&Ⅲ	94(52.22%)	72(60.00%)	

Abbreviations: AFP, alpha-fetoprotein; HBsAg, hepatitis B surface antigen; TNM, tumor node metastasis; BCLC, Barcelona Clinic Liver Cancer; CNLC, China Clinic Liver Cancer.

Supplementary Table S5. High expressed pathways in the Sema3d_Low group of Gene set enrichment analysis (GSEA)

NAME	NES	NOM p-val
**HALLMARK_PI3K_AKT_MTOR_SIGNALING**	**1.724282**	**0.005714**
HALLMARK_KRAS_SIGNALING_UP	1.597425	0.006085
HALLMARK_INFLAMMATORY_RESPONSE	1.586428	0.018256
HALLMARK_EPITHELIAL_MESENCHYMAL_TRANSITION	1.563879	0.027944
HALLMARK_APICAL_JUNCTION	1.554019	0.034205
HALLMARK_ALLOGRAFT_REJECTION	1.538897	0.046122
HALLMARK_APOPTOSIS	1.521574	0.047423
HALLMARK_IL2_STAT5_SIGNALING	1.513966	0.029598
HALLMARK_IL6_JAK_STAT3_SIGNALING	1.512123	0.036511
HALLMARK_ANGIOGENESIS	1.488971	0.049180

Supplementary Table S6. Low expressed pathways in the Sema3D_Low group of Gene set enrichment analysis (GSEA)

NAME	NES	NOM p-val
HALLMARK_OXIDATIVE_PHOSPHORYLATION	-1.80412	0.040698
HALLMARK_FATTY_ACID_METABOLISM	-1.08988	0.369388
HALLMARK_PEROXISOME	-1.04845	0.411157
HALLMARK_DNA_REPAIR	-0.95115	0.522088
HALLMARK_REACTIVE_OXYGEN_SPECIES_PATHWAY	-0.87475	0.614801
HALLMARK_ADIPOGENESIS	-0.84374	0.640657
HALLMARK_BILE_ACID_METABOLISM	-0.73125	0.748441
HALLMARK_XENOBIOTIC_METABOLISM	-0.65223	0.860082
HALLMARK_MYC_TARGETS_V1	-0.63144	0.757085
HALLMARK_MYC_TARGETS_V2	-0.51806	0.928425

Supplementary Table S7. Result of co-IP combined LC-MS/MS

Entry name	Coverage [%]	# PSMs	Score Sequest HT: Sequest HT
K2C1_HUMAN	59	74	221.85
K1C9_HUMAN	69	49	169.45
K22E_HUMAN	67	50	161.64
K1C10_HUMAN	52	35	117.44
**FLNA_HUMAN**	**12**	**30**	**85.13**
HSP7C_HUMAN	32	28	78.14
ALBU_HUMAN	31	26	70.7
ACTB_HUMAN	43	22	68.15
TBB4B_HUMAN	42	25	67.21
TBB5_HUMAN	44	23	63.66
HS90A_HUMAN	20	23	57.81
K2C6A_HUMAN	29	19	50.6
TBA1B_HUMAN	24	15	43.82
TBA1A_HUMAN	24	15	43.56
HS90B_HUMAN	21	18	43.17
G3P_HUMAN	50	14	41.06
BIP_HUMAN	19	11	39.18
H2A2C_HUMAN	30	14	38.35
K1C16_HUMAN	31	12	35.97
CH60_HUMAN	18	10	35.46
