# Supplementary material for: Sema3d Restrained Hepatocellular Carcinoma Progression Through Inactivating Pi3k/Akt Signaling via Interaction With FLNA
Source: Front Oncol. 2022 Jul 25;12:913498. doi: 10.3389/fonc.2022.913498 (PMC9358705; doi:10.3389/fonc.2022.913498)
Supplement: Supplementary file 3 [file DataSheet_3.pdf]

Supplementary methods and materials:

RNA extraction and quantitative real-time PCR

Total RNA was extracted from tissues or cells with TRIzol reagent (Invitrogen, Carlsbad, CA). Reverse transcription were performed using an Advantage RT-for-PCR Kit (Takara, Dalian, China). qRT-PCR analysis was done using SYBR®Green Real time PCR Master Mix assay kit (Takara) in a 7300 Real-Time PCR system (Applied Biosystems Inc., Foster City, CA) with the following primers: Sema3d forward, AGAAGAGTTCACAGCAGGTCT and reverse, ACAATGTTCTCTTTCAAATGGTGTT.

Transfection and clone selection

The ectopic expression and knockdown lentivirus as well as control lentivirus for Sema3d and FLNA were all purchased from GenePharma (Suzhou, China). Transfection was performed according to the manufacturer's instructions. Puromycin (2 μg/mL) was used to select stable clones. The sequences of shRNA are listed as follows: Sema3d-sh1: GCCGTCACTGAAGATGTAA AC; Sema3d-sh2: GCCAGTTCTACGGACAGAAGA; Sema3d-sh3: GCCCAGCAACGAATCAAATGG; FLNA-sh1: ATCAATTCAAGAGATTGATGT; FLNA-sh2: ACT TTTTCAAGAGAAAAGTTC; FLNA-sh3: CAATATTCA AGAGATATTGTT.

MTT assay

For MTT assay, 5×10^3 cells were seeded in 96-well plates, incubated for 0–7 days, MTT solution (10 μl of 2.5 mg/mL MTT, Thermo Fisher Scientific, USA) were added to the well under detection. The plates were incubated at 37°C for 4 h, followed by addition of 150 μl of dimethyl sulfoxide to each well, and then incubated at 37°C for another 10 min to dissolve the formazan crystals. The absorbance values were determined at 490 nm. The relative cell number was normalized by the absorbance from the control cells.

Survival analysis of TCGA dataset

The TCGA-LIHC dataset (n=425) and clinical data were obtained form with recurrence information from The Cancer Genome Atlas (TCGA, http://cancergenome.nih.gov/). For reducing the error, 79 cases were deleted which Sema3d mRNA expression or >50% genes expression value were missing. 16 cases were deleted which the survive time within 1 month, whose death mainly caused by the treatment but not tumor progression. Then the cases were grouped by median and the Kaplan-Meier method and log-rank test were performed to analyze the Overall survival, Disease free survival and Progression free Survival.

Gene set enrichment analysis (GSEA)

The GSEA (http://www.broadinstitute.org/gsea) tool was used to determine the differential enrichment of gene sets in the HCC patient samples belonging to high and low Sema3d expression groups. Gene expression data from TCGA were divided into high and low expression groups according to the median level of Sema3d mRNA expression, and GSEA was used to explore the influence of Sema3d expression level on each gene and to analyze the mechanism underlying the involvement of Sema3d in the invasion and metastasis of HCC. The genome was sequenced 1000 times per analysis. In addition, the level of Sema3d was used as a phenotypic marker. The nominal p-value (NOM p) and the normalized enrichment score (NES) were used to classify enrichment

pathways in each phenotype.

Co-immunoprecipitation (co-IP) assay

For Co-IP, pre-cleared protein from whole cell lysates were incubated with antibody against Sema3d or FLNA at 4 °C overnight, which was conjugated to AminoLink Plus Resin (Pierce, Rockford, IL), The IP targets were disassociated from the immobilized antibodies on the AminoLink Plus Resin by the gentle elution buffer. Eluted proteins were resolved using 10% SDS-PAGE, followed by western blot with appropriate antibodies.

Cell immunofluorescence staining

Indicated HCC cells (2×10^4 cells) were seeded into 12-well plate with glass coverslips for 24 h. Then cells were successively fixed in 4% paraformaldehyde, permeabilized with 0.25% Triton X-100, blocked with 1% BSA and incubated with primary antibody at 4 °C overnight. After washed with PBS, cells were incubated with appropriate DyLight-conjugated secondary antibody and DAPI (Vector laboratories, Burlingame, CA). Finally, the slides were mounted and images were captured using an inverted fluorescence microscope DMI4000-B (Leica, Wetzlar, Germany).

Table. 1 The sequences of PCR primers used in this study

Genes	Application	Sequence (5’→3’)
Sema3d	qRT-PCR	F: AGAAGAGTTCACAGCAGGTCT
		R: ACAATGTTCTCTTTCAAATGGTGTT
GAPDH	qRT-PCR	F: TGGCCTTCCGTGTTCCTAC
		R: GAGTTGCTGTTGAAGTCGCA
FLNA	qRT-PCR	F: GGGTCCAAGTCCAGGACAAT
		R: CTGCCAGCTCCCACATTCA
E-cadherin	qRT-PCR	F: AGTGACTGATGCTGATGCCC
		R: AATGTACTGCTGCTTGGCCT
Vimentin	qRT-PCR	F: AGAGGAAGCCGAAAACACCC
		R: GCTCCTGGATTTCCTCTTCG
Snail	qRT-PCR	F: GCAGGACTCTAATCCAGAGTTTA
		R: CCACAGAAATGGCCATGGGA

Table.2. The primary antibodies used in this study

Primary Antibodies	Origin	Application	Dilution
Sema3d	Abcam (ab180174)	IHC	1:50
		WB	1:500
		IP	1:300

		IF	1:200
FLNA	Abcam (26205-1-AP)	IHC	1:50
		IF	1:200
		WB	1:500
p-FLNA	Abcam(ab51229)	WB	1:500
C-FLNA	Abcam(ab238514,C terminal)	WB	1:500
		IF	1:200
E-cadherin	CST (#14472)	WB	1:1000
		IF	1:200
		IHC	1:100
Vimentin	CST (#5741)	WB	1:1000
		IF	1:100
		IHC	1:200
Snail	CST(#3895)	WB	1:500
Pi3k	CST(#34050)	WB	1:500
p-Pi3k	CST(#17366)	WB	1:500
Akt	CST (#14702)	WB	1:500
p-Akt	CST (#4058)	WB	1:500
β-actin	Affinity (#T0022)	WB	1:2000
GAPDH	Affinity (AF0863)	WB	1:1000

Table.3 List of secondary antibodies and reagents used in this study

**Secondary antibodies or reagents**	**Origin**	**Application**	**Dilution**
Goat anti-Rabbit IgG (H+L) Secondary Antibody, DyLight 594 conjugate	ThermoFisher Scientific (#35560)	IF	1:100
Goat anti-Rabbit IgG (H+L) Secondary Antibody, DyLight 488 conjugate	ThermoFisher Scientific (#35553)	IF	1:100

Goat anti-Mouse IgG (H+L) Secondary Antibody, DyLight 594 conjugate	ThermoFisher Scientific (#35510)	IF	1:100
Goat anti-Mouse IgG (H+L) Secondary Antibody, DyLight 488 conjugate	ThermoFisher Scientific (#35503)	IF	1:100
Mitomycin C	Roche (M8170)	/	10 μg/ml
Wortmannin	Selleck(S2758)	/	2.5 μM
Phalloidin	US Everbright(YP0052)	IF	200U/ml
DMSO	Solarbio(D8371)	Solvent	/

Table. 4. The sequences of RNAi used in this study

Name	Sequence
Sema3d -shRNA1	5’- GCCGTCACTGAAGATGTAA AC -3’
Sema3d -shRNA2	5’- GCCAGTTCTACGGACAGAAGA -3’
Sema3d -shRNA3	5’- GCCCAGCAACGAATCAAATGG -3’
FLNA -shRNA1	5′ - ATCAATTCAAGAGATTGATGT -3′
FLNA -shRNA2	5′ - ACT TTTTCAAGAGAAAAGTTC -3′
FLNA -shRNA3	5′ - CAATATTCA AGAGATATTGTT -3′
