# Supplementary figures and images for: Sema3d Restrained Hepatocellular Carcinoma Progression Through Inactivating Pi3k/Akt Signaling via Interaction With FLNA
Source: Front Oncol. 2022 Jul 25;12:913498. doi: 10.3389/fonc.2022.913498 (PMC9358705; doi:10.3389/fonc.2022.913498)

Fig1C

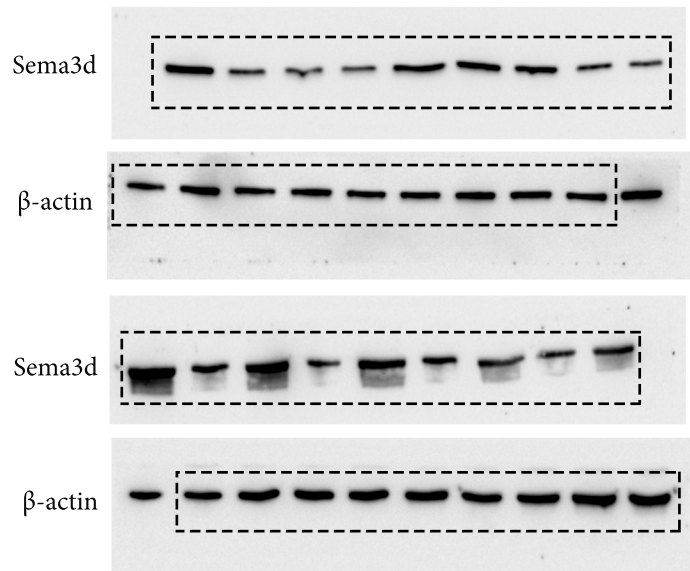

Fig4B

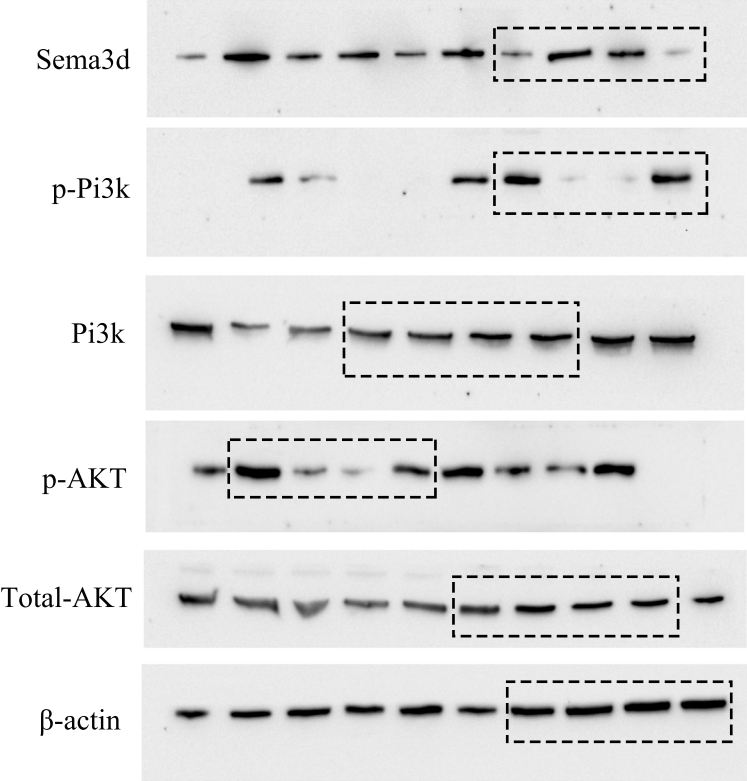

Fig5B

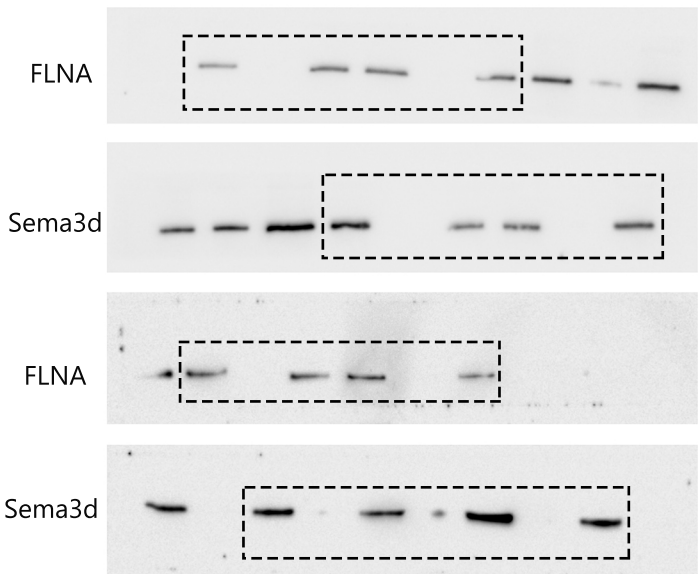

Fig5D

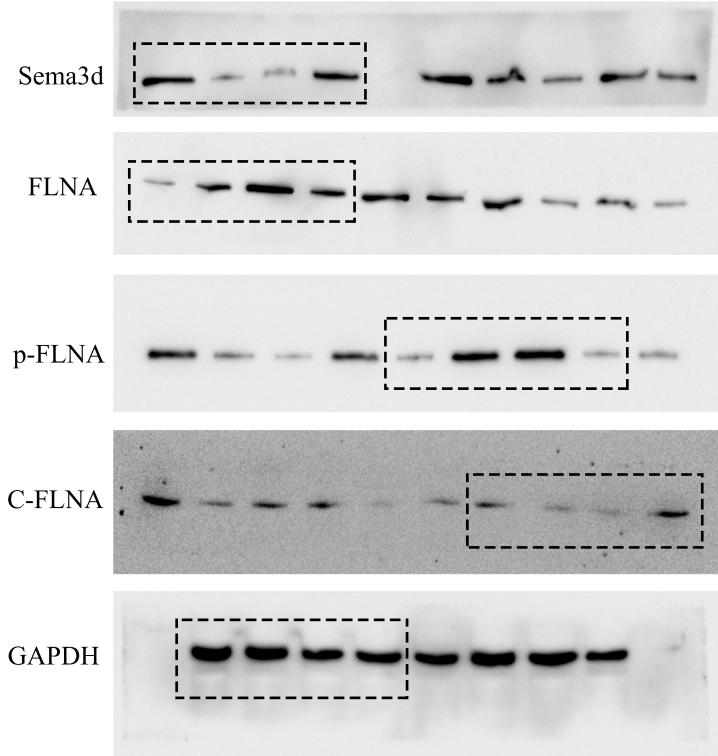

Supplement: Supplementary file 4 [file DataSheet_4.pdf]

Fig6C

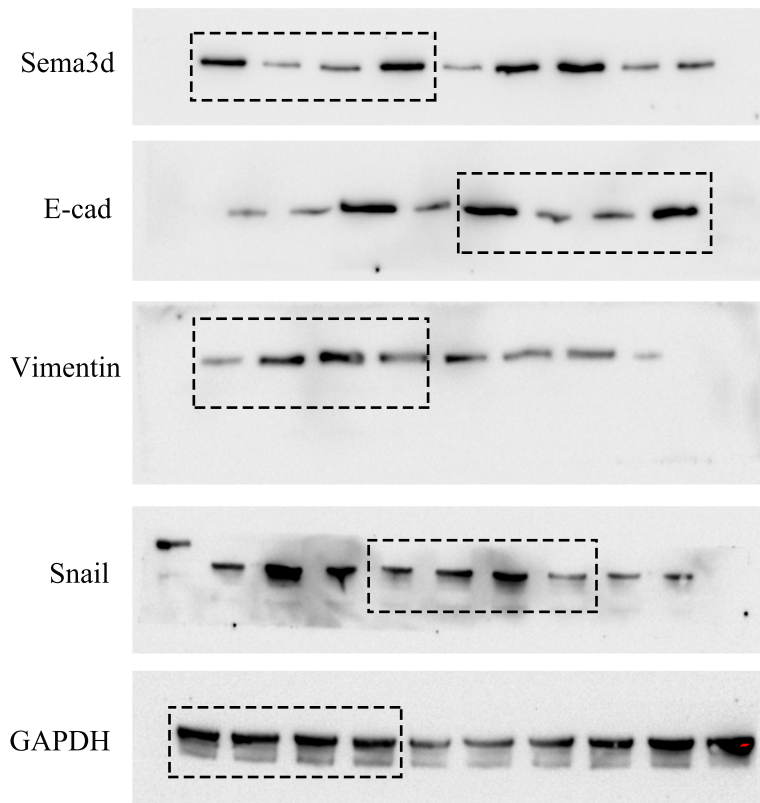

Fig7A

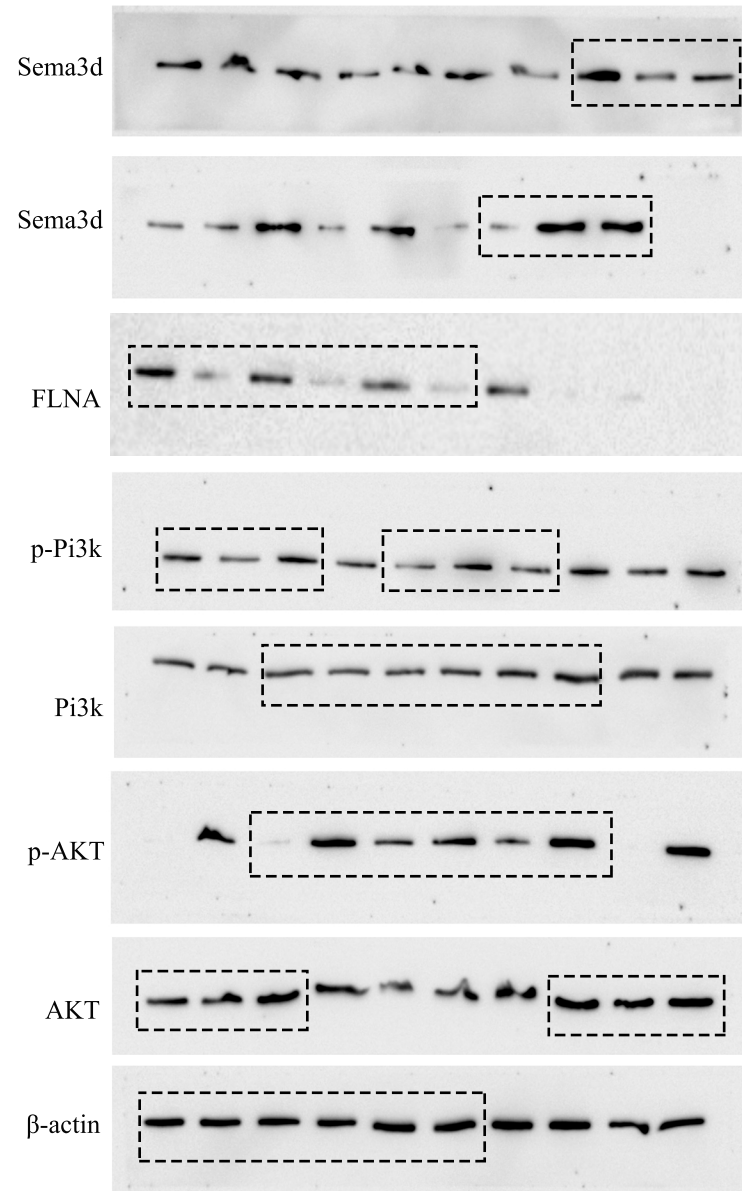

FigS3

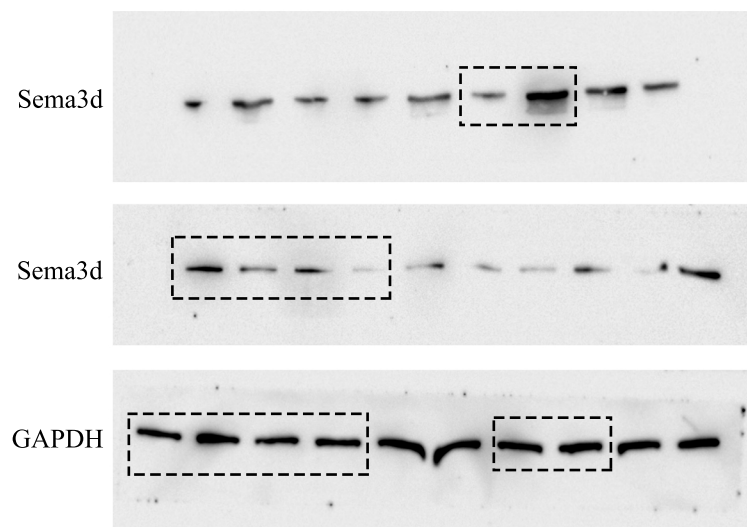

Fig7A

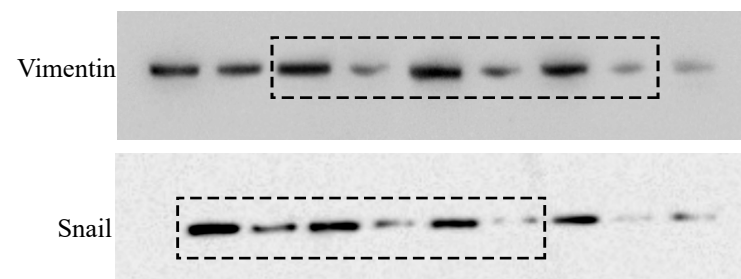

Supplement: Supplementary file 5 [file DataSheet_5.pdf]
